# Peptide recognition by a synthetic receptor at subnanomolar concentrations†

**DOI:** 10.1039/d4sc01122h

**Published:** 2024-03-12

**Authors:** Paolo Suating, Marc B. Ewe, Lauren B. Kimberly, Hadi D. Arman, Daniel J. Wherritt, Adam R. Urbach

**Affiliations:** a Department of Chemistry, Trinity University 1 Trinity Place San Antonio TX 78212 USA aurbach@trinity.edu; b Department of Chemistry, University of Texas at San Antonio 1 UTSA Circle San Antonio TX 78249 USA

## Abstract

This paper describes the discovery and characterization of a dipeptide sequence, Lys–Phe, that binds to the synthetic receptor cucurbit[8]uril (Q8) in neutral aqueous solution with subnanomolar affinity when located at the N-terminus. The thermodynamic and structural basis for the binding of Q8 to a series of four pentapeptides was characterized by isothermal titration calorimetry, NMR spectroscopy, and X-ray crystallography. Submicromolar binding affinity was observed for the peptides Phe-Lys-Gly-Gly-Tyr (FKGGY, 0.3 μM) and Tyr-Leu-Gly-Gly-Gly (YLGGG, 0.2 μM), whereas the corresponding sequence isomers Lys-Phe-Gly-Gly-Tyr (KFGGY, 0.3 nM) and Leu-Tyr-Gly-Gly-Gly (LYGGG, 1.2 nM) bound to Q8 with 1000-fold and 170-fold increases in affinity, respectively. To our knowledge, these are the highest affinities reported between a synthetic receptor and an unmodified peptide. The high-resolution crystal structures of the Q8·Tyr-Leu-Gly-Gly-Gly and Q8·Leu-Tyr-Gly-Gly-Gly complexes have enabled a detailed analysis of the structural determinants for molecular recognition. The high affinity, sequence-selectivity, minimal size of the target binding site, reversibility in the presence of a competitive guest, compatibility with aqueous media, and low toxicity of Q8 should aid in the development of applications involving low concentrations of target polypeptides.

## Abstract 2

The synthetic receptor cucurbit[8]uril (Q8) binds the N-terminal dipeptide site Lys-Phe with subnanomolar affinity in neutral aqueous buffer.

## Graphical abstract

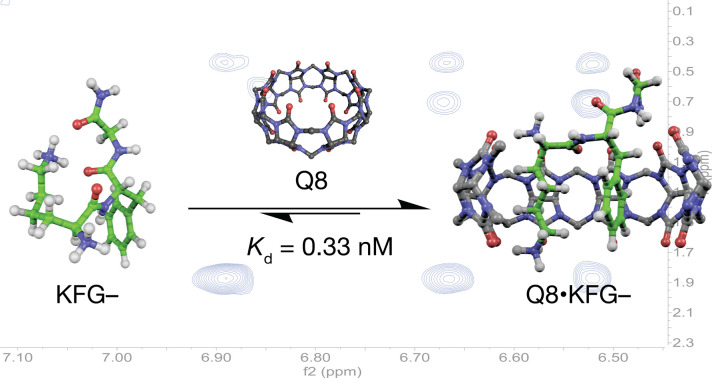

## Introduction

Biomedical science relies on methods for adding ligand binding sites to proteins. Such affinity tags are essential for protein affinity purification, detecting and quantifying proteins of interest, improving protein solubility, and adding functionality to proteins such as fluorescent labels or sites for chemical modification.^1–4^ Protein affinity tags are often incorporated *via* recombinant DNA methodology and may comprise large fusion domains such as glutathione-*S*-transferase and maltose-binding protein, or small (5–20 residue) oligopeptide epitope tags such as His, Myc, and FLAG tags.^4–7^ Although affinity tags assist greatly in purification, they often need to be removed afterward due to unwanted influence on the structure and/or function of the target protein. For example, His tags add several positive charges. Removal of the tag (*e.g.*, *via* endopeptidase cleavage) and subsequent purification leads to considerable loss of material. We posit that an affinity tag comprising only two residues should impose minimally on protein structure/function and therefore reduce the likelihood that the tag would need to be removed.

In order to be useful in biological applications, oligopeptide affinity tags must be recognized with high affinity and sequence-selectivity by their cognate receptor. Creating minimal affinity tags is especially challenging due to the limited structural information and surface area inherent to a small target site. To date, the smallest oligopeptide affinity tags are 4–6 residues in length and comprise metal chelators (*e.g.*, oligo-His), polyionic tags for ion-exchange chromatography (*e.g.*, oligo-Glu and oligo-Arg), or tags recognized sequence-selectively by an antibody (*e.g.*, C-tag).^4^ In general, sequence-selectivity has been challenging to achieve with synthetic ligands. The most promising ligands to date include molecularly imprinted polymers (MIPs) and synthetic receptors. MIPs are polymeric nanoparticles formed by templating a binding cavity into a crosslinked polymer using specific epitopes.^8^ MIPs can be highly selective for their targets and can have sub-nanomolar detection limits for large proteins, but the affinities of MIPs for oligopeptide targets are limited to the high nanomolar range.^9^ Moreover, the development of MIPs for biological applications is hindered by their heterogeneous compositions and limited molecular characterization. Synthetic receptors, by contrast, are pure organic compounds, typically macrocycles, that are highly stable and relatively inexpensive but, with the exception of cucurbit[n]urils, lack the affinities and selectivities necessary to be useful in many biological applications. The present work aims to make progress toward overcoming these challenges.^9^

**Fig. 1:**
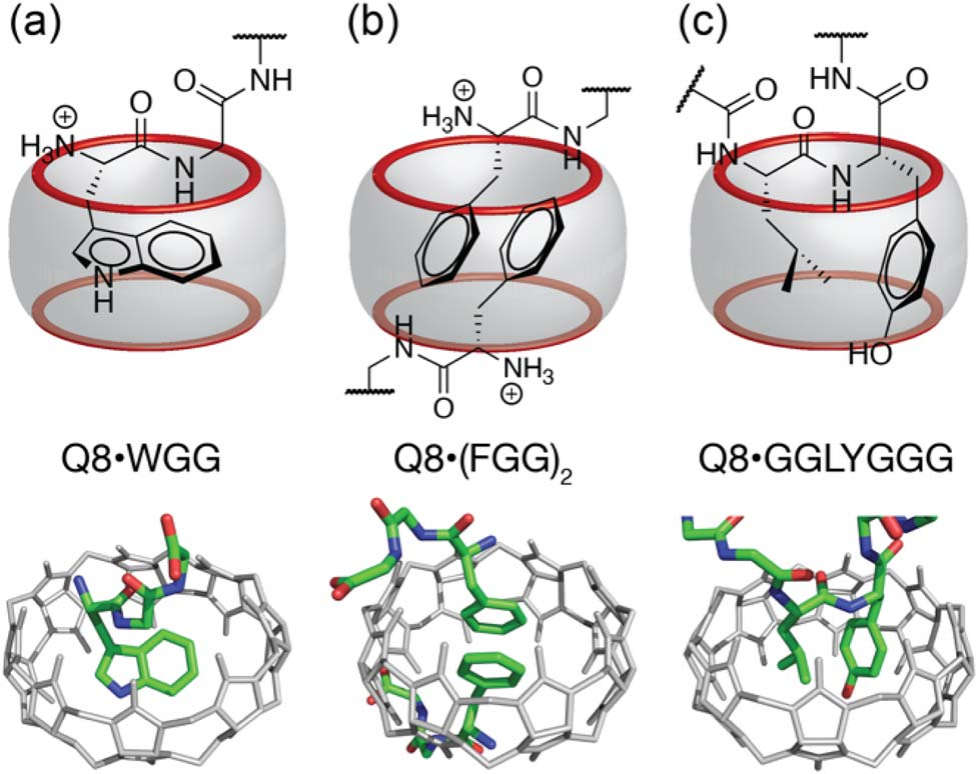
Three unique binding motifs of Q8·peptide complexes, with schematics on top and crystal structures on bottom. (a) The Q8·WGG complex shows recognition of N-terminal Trp on a single peptide, with the indole side chain bound within the Q8 cavity and chelation of the N-terminal ammonium group by three C=O oxygens; (b) the Q8·(FGG)_2_ complex shows recognition of the N-terminal Phe on each of two peptides, with staggered face-to-face stacking of the benzyl side chains and binding of each N-terminal ammonium group at opposite Q8 portals. (c) the Q8·GGLYGGG complex shows recognition of the Leu–Tyr dipeptide site, with inclusion of both side chains and folding of the peptide backbone into a type II β-turn. Host molecules are shown in gray, and peptides are colored as follows: carbon in green, nitrogen in blue, and oxygen in red. Hydrogen atoms and water molecules were removed for clarity.

Cucurbit[*n*]urils are a family of synthetic, barrel-shaped receptors comprising *n* repeating glycoluril units bridged by 2*n* methylene groups.^10,11^ These rigid macrocylic hosts have a weakly hydrated inner cavity that is accessible *via* two symmetric portals, each lined by *n* ureido carbonyl groups. This structure creates a ring of negative electrostatic potential at the constricted portals, which, in combination with the weakly hydrated cavity, is well-suited to guests containing both nonpolar and cationic groups. Prior work has shown that cucurbit[7]uril (Q7) and cucurbit[8]uril (Q8) can bind oligopeptides sequence-selectively and with sub-millimolar to mid-nanomolar binding affinities.^12–21^ Q7 and Q8 recognize the aromatic residues Phe (F), Tyr (Y), and Trp (W) by inclusion of the aromatic side chain within the Q*n* cavity (Fig. 1a). Selectivity for the N-terminal position is mediated by electrostatic attraction between the N-terminal ammonium group and C=O groups on the Q*n*. While Q7 has a cavity volume sufficient to accommodate a single side chain, the larger cavity of Q8 can accommodate the side chains of up to two residues. Q8 can noncovalently dimerize two peptides by including a single aromatic residue from each peptide (Fig. 1b).^14^ Q8 can also bind to two neighboring residues on a single peptide. In the latter case, known as the “pair-inclusion motif,” the peptide backbone folds in order to accommodate the simultaneous inclusion of the pair of neighboring side chains (Fig. 1c).^19–22^ At non-terminal sites, the backbone folds into a type II β-turn.^23^

Numerous applications have derived from the sequence-selective recognition of peptides by Q7 and Q8, including peptide sensing,^12,19,24–27^ peptide and cell capture and release,^28–30^ enhancing peptide detection,^31^ inhibiting and measuring protease activity,^32,33^ and supramolecular polymers and hydrogels.^34–38^ Recognition properties are retained when oligopeptide epitopes for Q7 and Q8 are incorporated onto larger proteins,^17^ enabling applications including protein dimerization,^39–41^ oligomerization,^42^ and polymerization,^43,44^ modulation of protein aggregation,^45,46^ protein capture on surfaces,^47,48^ and protein drug formulation.^49,50^ These applications were aided by the biocompatibility, commercial availability, and stability (thermal, chemical, and metabolic) of Q7 and Q8, the derivatization and conjugation strategies of Q7, the predictable and well-understood sequence-selectivity of these systems, and the reversibility of binding using small molecule competitors across a wide range of binding affinities.^11,51^

Applications of cucurbit[*n*]uril-oligopeptide interactions can be limited, however, by binding affinity. In order to target the many peptides and proteins that exist in biological systems at or below the nanomolar concentration range, it is necessary for the equilibrium dissociation constants to be at or below the nanomolar range. Although there have been a few reports of *K*_d_ values in the nanomolar range,^15,18–21,23^ to the best of our knowledge there have been no reports to date of sub-nanomolar binding of unmodified peptides by synthetic receptors. We recently investigated the binding of Q8 to non-terminal dipeptide sites.^23^ Although binding was expected to be weaker at non-terminal sites due to the lack of involvement of the N-terminal ammonium group, we discovered that Q8 binds to non-terminal KF and FK dipeptide sites in the pair-inclusion motif with *K*_d_ values of 60 and 86 nM, respectively. These affinities were higher than that of non-terminal YL and LY, which were the highest affinity sites observed in previous work. We hypothesized that moving the KF and FK sites to the N-terminus could yield higher binding affinity than previously observed.

## Results and discussion

### Thermodynamic studies

Two pentapeptides, H-Lys-Phe-Gly-Gly-Tyr-OH (KFGGY) and H-Phe-Lys-Gly-Gly-Tyr-OH (FKGGY), were designed to contain the target dipeptide site (bold) at the N-terminus and were obtained commercially (see ESI† for experimental details). C-terminal Tyr was included to aid in quantitation by UV spectroscopy. The thermodynamics of complexation for Q8 binding with the two peptides were determined using isothermal titration calorimetry at 300 K in 10 mM sodium phosphate, pH 7.0 (Table 1 and Fig. S3–S5†). Direct titration of Q8 with KFGGY resulted in a steep binding curve (Fig. S3†), suggestive of an affinity that is too strong to be determined confidently by ITC.^52^ Therefore, a competition binding experiment was performed by ITC using methyl viologen (MV, *N*,*N*′-dimethyl-4,4′-bipyridinium dichloride) as the weak competitor in 100-fold stoichiometric excess of Q8 (Fig. S4†), as described previously.^18,53^ This experiment yielded a *K*_d_ value of 0.33 (±0.08) nM. To our knowledge, this is the highest affinity observed between a synthetic receptor and an unmodified peptide. In order to increase confidence in this result, we also measured the *K*_d_ value for the Q8·KFGGY complex by the NMR competition method reported by Isaacs and co-workers (Fig. S16†).^51^ Using (ferrocenylmethyl)trimethylammonium chloride (FcNMe_3_) as the competitor (Fig. S1 and S2†), we determined a *K*_d_ value of 0.27 (±0.05) nM, which is very similar to the value determined by competitive ITC. Interestingly, the sequence isomer, FKGGY, bound Q8 with *K*_d_ value of 330 nM, which is three orders of magnitude weaker in affinity. When KF and FK dipeptide sites were located at non-terminal positions, their affinities for Q8 differed by less than 2-fold.^23^

**Table 1:** Thermodynamic binding data for Q8·peptide complexes

Guest	*K*_d_ (nM)	Δ*H* (kcal mol^−1^)	−*T*Δ*S* (kcal mol^−1^)
KFGGY	0.33 ± 0.08^a^	−16.6 ± 0.4^a^	3.6 ± 0.3^a^
KFGGY	0.27 ± 0.05^b^	nd	nd
FKGGY	330 ± 40^c^	−13.9 ± 0.4^c^	5.0 ± 0.3^c^
LYGGG	1.2 ± 0.1^a^	−17.0 ± 0.1^a^	4.7 ± 0.1^a^
YLGGG	150 ± 20^c^	−14.9 ± 0.2^c^	5.5 ± 0.2^c^

a Average values (± standard deviations) for data collected in triplicate *via* ITC competition experiments using methyl viologen dichloride (MV) at a 1 : 100 ratio of Q8 : MV. *K*_d_ (MV) = 6.8 (±0.2) × 10^−7^ M; Δ*H* (MV) = −4.8 (±0.2) kcal mol^−1^; −*T*Δ*S* (MV) = −3.7 ± 0.1 kcal mol^−1^.

b Average values (± standard deviations) for data collected in triplicate *via* NMR competition experiments with (ferrocenylmethyl)trimethylammonium. The affinity of FcNMe_3_^+^ was determined by ITC competition with MV. *K*_d_ (FcNMe_3_) = (4.4 ± 0.7) × 10^−10^ M.

c Average values (± standard deviations) for data collected in triplicate *via* ITC with direct titration of guest to host. nd = not determined.

In our first publication on the pair-inclusion motif,^19^ we reported the *K*_d_ value for Q8 in complex with the tripeptide H-Tyr-Leu-Ala-NH_2_ as 8 nM using competitive ITC. Scherman and coworkers also studied the YL dipeptide binding site and reported *K*_d_ values for Q8 binding to H-Tyr-Leu-Ala-NH_2_ (120 nM), H-Tyr-Leu-NH_2_ (120 nM), and H-Tyr-Leu-Ala-Ala-NH_2_ (140 nM) using ITC by direct titration.^22,23^ Puzzled by these reports, we revisited the H-Tyr-Leu-Ala-NH_2_ and agree that the binding constant is closer to 100 nM than 10 nM. This is important because YL has been the benchmark target dipeptide site in four studies on the pair-inclusion motif,^19,21–23^ and similarly we wanted to compare the binding affinities observed for KFGGY and FKGGY to the YL benchmark. Therefore, two additional pentapeptides, H-Tyr-Leu-Gly-Gly-Gly-OH (YLGGG) and H-Leu-Tyr-Gly-Gly-Gly-OH (LYGGG) were obtained commercially and characterized by ITC (Table 1, Fig. S6–S8†). We chose the buffer condition of 10 mM sodium phosphate, pH 7.0, to allow direct comparison to prior work on Q8·peptide molecular recognition. YLGGG was found to bind Q8 with a *K*_d_ value of 150 nM, as determined by direct titration, whereas its sequence isomer, LYGGG, bound ∼100-fold more tightly (1.2 nM), as determined by competitive titration using MV as competitor. In all cases, binding is enthalpically driven and entropically unfavourable. For each peptide with an aliphatic N-terminal residue and aromatic 2nd residue (*i.e.*, KFGGY and LYGGG), binding affinity was stronger, more exothermic, and less entropically unfavourable than for its sequence isomer (*i.e.*, FKGGY and YLGGG, respectively). The three-fold difference in binding affinity between FKGGY and YLGGG shows a lack of selectivity between these two sites. ITC and mass spectrometry data corroborate the Q8 : peptide binding stoichiometry of 1 : 1 in all cases (Fig. S20–S27†).

### Structural studies by ^1^H NMR spectroscopy

Spectra were collected at 500 MHz at 21 °C on samples dissolved in 10 mM sodium phosphate-buffered D_2_O, pH_apparent_ 7.1.^54^ Upon addition of a substoichiometric quantity of Q8 to KFGGY or FKGGY, we observed the appearance of a second set of peptide signals, with each set corresponding to either the free peptide or the peptide in the 1 : 1 complex (Fig. S9 and S17†). This result indicates slow chemical exchange on the NMR time scale and is consistent with other studies on the pair-inclusion motif. In the second set of peaks, we observed a perturbation to lower chemical shift values of the signals corresponding to the Lys and Phe side chains, indicating the shielding of the side chains from the external magnetic field due to binding within the Q8 cavity.^55–57^ The signals corresponding to the Tyr side chain do not perturb upon the addition of Q8, showing that Tyr does not interact significantly with the Q8 cavity or portals.

^1^H–^1^H correlation spectroscopy (COSY) was used to assign the signals of the Lys sidechain (Fig. S10 and S13†) in the spectra for KFGGY. In the bound state, the geminal protons were resolved for all methylene groups in the chain except δ-CH_2_. Peak separations for geminal pairs were observed to be as small as 0.1 ppm in the case of β-CH_2_ and as large as 0.8 ppm in the case of γ-CH_2_. The resolution of signals for the ε-CH_2_ geminal protons is likely aided by their proximity to the Phe aromatic side chain.

**Fig. 2:**
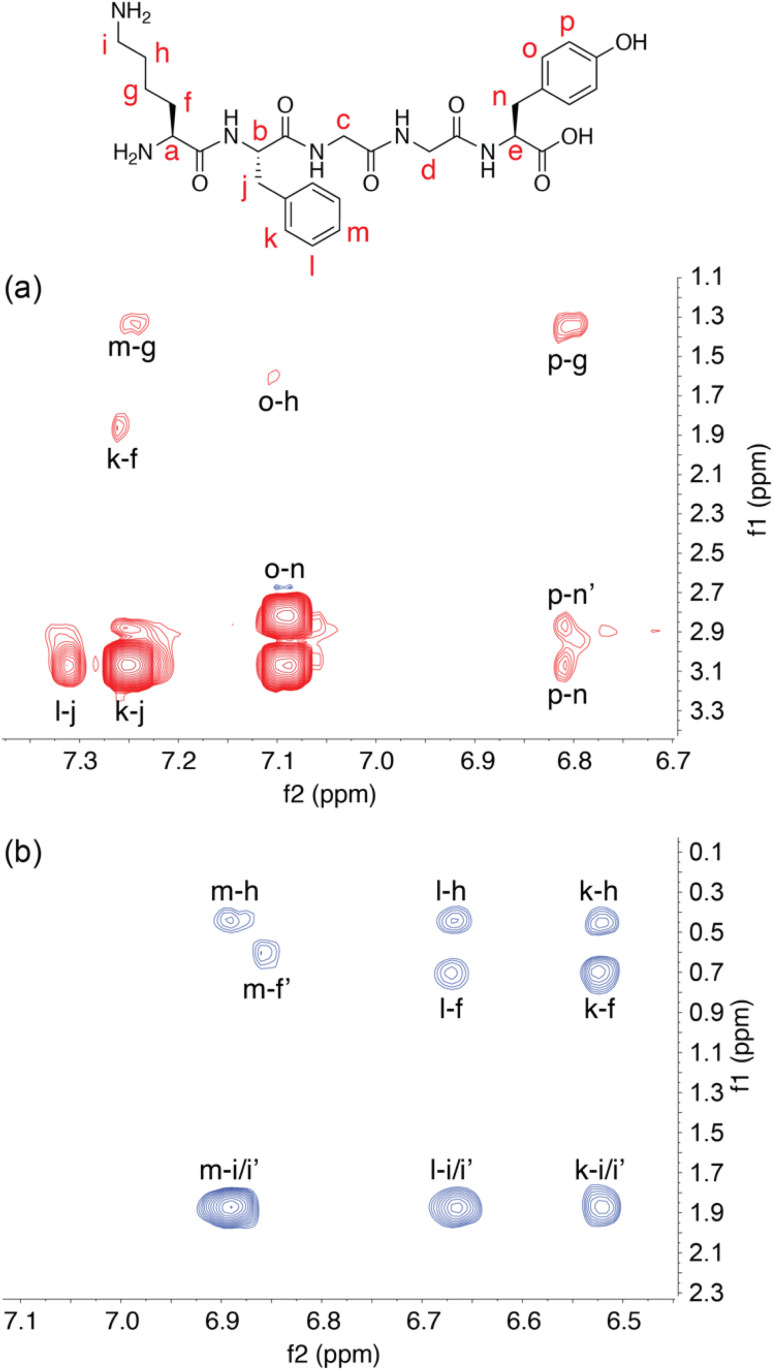
Two-dimensional ^1^H NMR spectroscopy. (a) Aromatic–aliphatic region of a 2-D NOESY spectrum of free KFGGY, showing cross peaks between the side chains of the Lys, Phe, and Tyr residues. (b) The same region of a 2-D ROESY spectrum of KFGGY in complex with Q8, showing only cross peaks between the side chains of Lys and Phe residues. Letters are used to correlate hydrogens on the peptide structural formula with corresponding signals.

To further investigate the solution structure of the Q8·KFGGY complex, 2-D ^1^H–^1^H Nuclear Overhauser Effect Spectroscopy (NOESY) data were collected and analysed for the free peptide, whereas the Q8·peptide complex was analysed by 2-D ^1^H–^1^H rotating-frame NOESY (*i.e.*, ROESY) (Fig. 2). In the unbound state, we observed cross-peaks between the signals corresponding to the side chains of Lys, Phe, and Tyr, for example Phe-*para* with Lys-γ, and Tyr-*meta* with Lys-γ. These NOEs suggest that on the NMR time scale the solution-state free peptide exists, on average, in a somewhat stably folded state. In the presence of Q8, however, we did not observe those inter-side-chain NOEs. Instead, in the presence of Q8 we observed cross-peaks correlating all Phe *ortho*-, *meta*-, and *para*- protons with ε-Lys, δ-Lys, and β-Lys protons. This result, in combination with the upfield chemical shift perturbation of the Phe and Lys side chains, is consistent with the proximity of Lys and Phe side chains within the Q8 cavity and the position of the C-terminal Tyr distal to the binding site. It is also clear that the Q8-induced fold of the peptide is different than the fold adopted in the absence of Q8. This result is also consistent with a cation–π interaction between the Lys ε-NH_3_^+^ group and the Phe aromatic ring, a motif that is commonly observed in protein crystal structures.^58–61^ The magnetic field anisotropy of the aromatic ring imparts additional shielding on the Lys methylene groups beyond what is induced by binding within the host cavity.^62^ This is evident not only in the differentiation of the diastereotopic protons in Lys, but also in the large perturbation of the Lys methylene signals to lower chemical shift values upon Q8 binding.

### Structural studies by X-ray crystallography

Further structural investigation was carried out by X-ray crystallography. The poor solubility of the complexes of Q8 with LYGGG and YLGGG impeded analysis by ^1^H NMR spectroscopy but facilitated the growth of single co-crystals for analysis by X-ray crystallography. Plate-like co-crystals of the Q8·LYGGG and Q8·YLGGG complexes were grown from approximately equimolar mixtures of host and guest at 3–4 mM concentration in 10 mM sodium phosphate-buffered D_2_O, pH_apparent_ 7.1. Crystal structures were obtained at resolutions of 0.82 Å for Q8·LYGGG and 0.79 Å for Q8·YLGGG (Tables S1 and S2,† CCDC 2314758 and 2313004). The Q8·YLGGG complex crystallized in the *P*2_1_ space group, with one Q8·YLGGG complex and eight associated water molecules in the asymmetric unit and two asymmetric units per unit cell. The Q8·LYGGG complex crystallized in the *C*_2_ space group, with one Q8·LYGGG complex and 44 associated water molecules in the asymmetric unit and one asymmetric unit per unit cell. All of the observed water molecules for the Q8·LYGGG complex are located outside of the Q8 cavity.

**Fig. 3:**
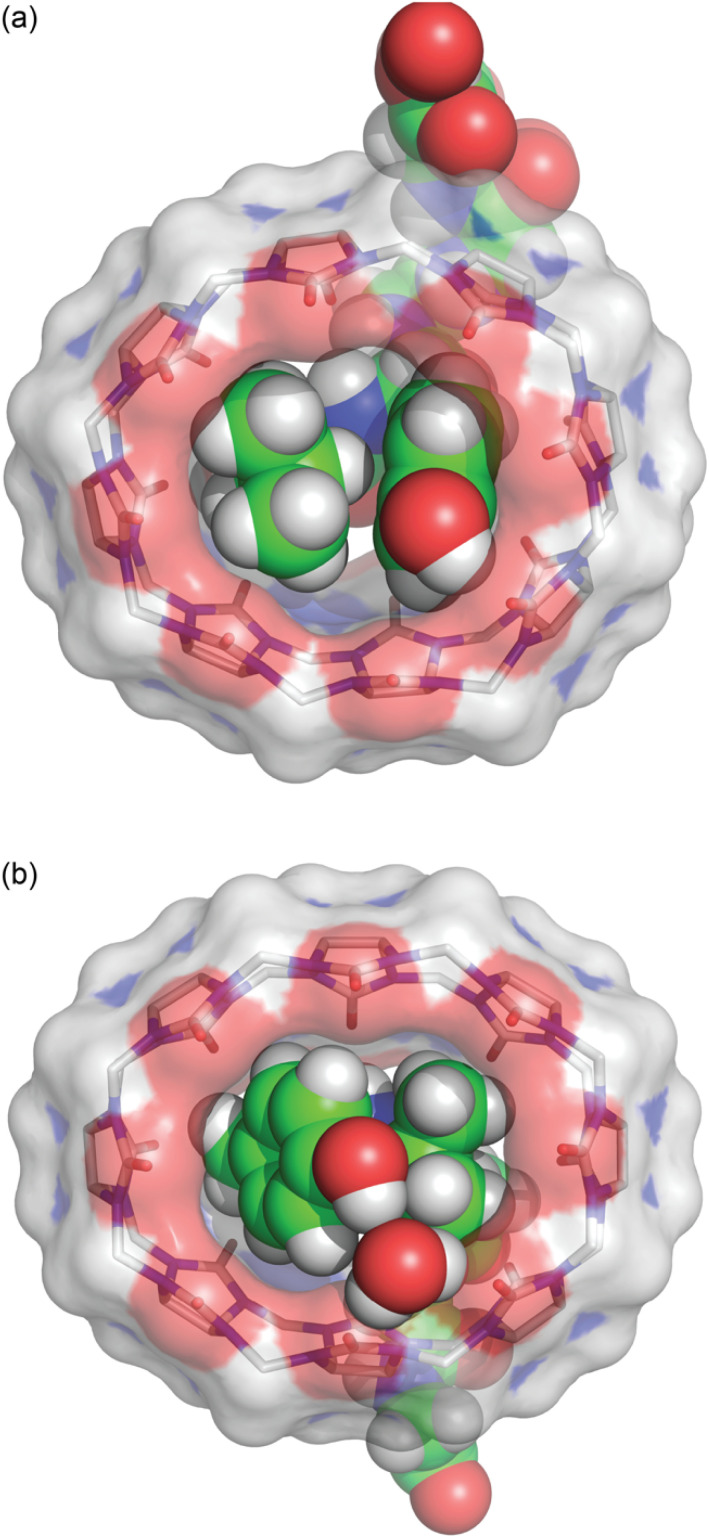
Bottom view of the crystal structures of (a) Q8·LYGGG and (b) Q8·YLGGG. The inclusion of Leu and Tyr side chains is apparent in both structures, as is the electrostatic interaction between the Tyr phenolic OH group and the Q8 portal, either *via* direct hydrogen bond (Q8·LYGGG) or *via* a bridging water molecule (Q8·YLGGG). The shape of the host is considerably puckered in the Q8·YLGGG complex but less so in the Q8·LYGGG complex. Host molecules are rendered as sticks with transparent solvent-accessible surface, and peptides and the bridging water are rendered as space-filling models. The molecules are coloured as follows: Q8 carbons in grey, peptide carbons in green, nitrogens in blue, oxygens in red, and hydrogens in white.

In both structures, Q8 is deformed from the ideal *D*_8h_ symmetry observed in its un-complexed state^63^ to a distorted ellipse, with greater deformation and a small degree of twisting observed in the Q8·YLGGG complex (Fig. 3). In both structures the side chains of Tyr and Leu residues are included within the Q8 cavity, and there is a CH–π interaction between the Leu γ-CH and the centre of the electron-rich Tyr phenol ring,^64,65^ as expected in this motif.^19,23^ The Leu γ-CH is more closely aligned with the centre of the Tyr phenol ring in the Q8·LYGGG structure.

**Fig. 4:**
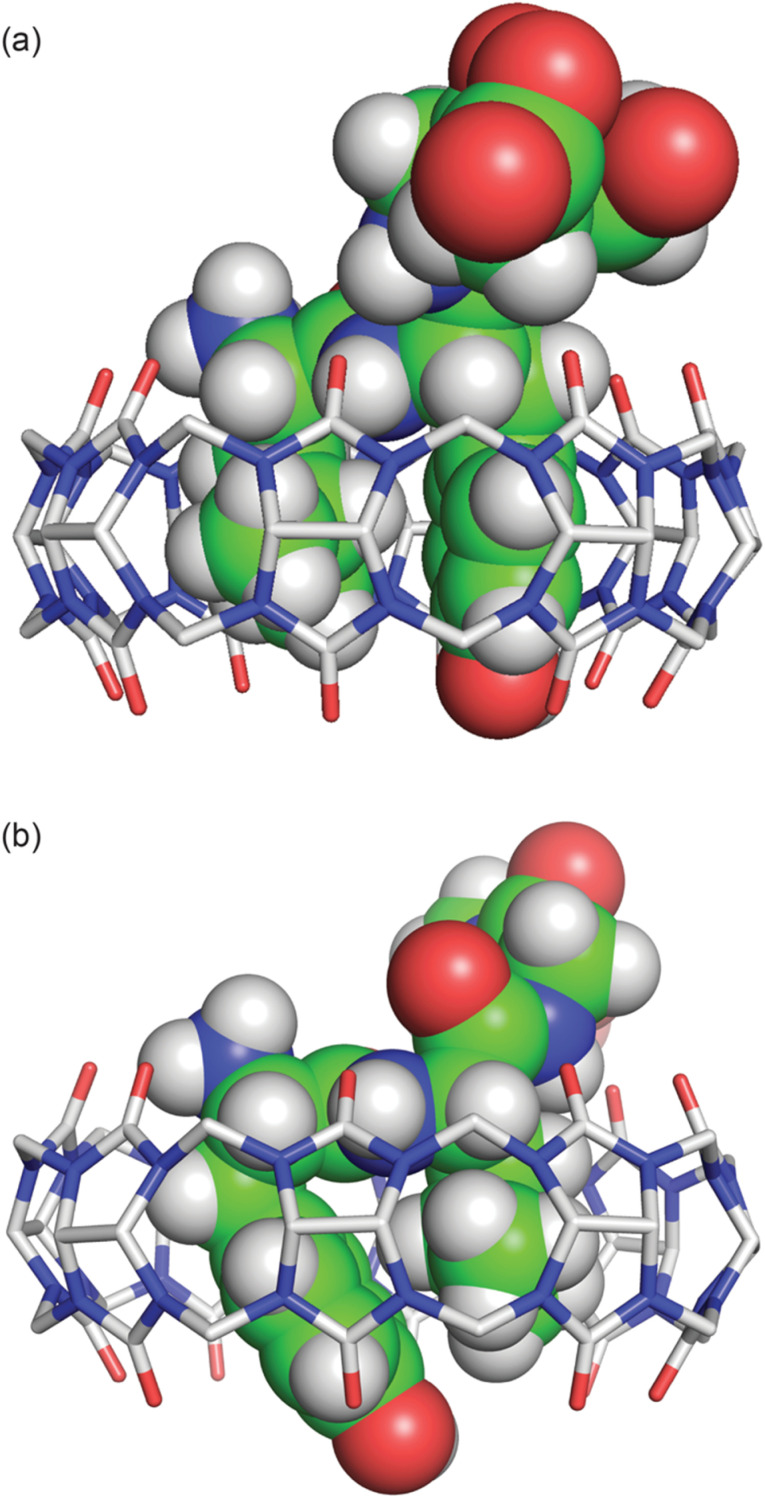
Side view of the crystal structures of (a) Q8·LYGGG and (b) Q8·YLGGG. In both structures, the N-terminal ammonium group is proximal to the Q8 portal, and the Leu and Tyr side chains are closely stacked and buried within the Q8 cavity. The peptide is buried more deeply in the Q8·YLGGG structure than in the Q8·LYGGG structure. The Tyr side chain of YLGGG is projected at an angle, positioning the phenolic OH group just outside the centre of the Q8 portal, too far to form a direct hydrogen bond. Host molecules are rendered as sticks with hydrogens omitted for clarity, and peptides are rendered as space-filling models with crystallographic disorder removed. The molecules are coloured as follows: Q8 carbons in grey, peptide carbons in green, nitrogens in blue, oxygens in red, and hydrogens in white.

The peptide bond between the Tyr and Leu residues is positioned such that the Tyr–Leu peptide bond of YLGGG is coplanar with the ring of carbonyl oxygens of the host portal (Fig. 4), which is similar to that observed in the crystal structure of Q8 in complex with GGLYGGG.^23^ In the Q8·LYGGG structure, however, the plane of the Leu–Tyr peptide bond is positioned at a 30° dihedral angle with respect to the ring of the Q8 carbonyl oxygens, with the amide NH group canted inward toward the Q8 cavity. In both structures, the peptide folds in the same direction, in which the α–CH bond of the N-terminal residue is anti-coplanar with the vicinal C=O bond. This fold is similar to our semiempirical structure of the Q8·YLA complex^19^ and similar to a model subsequently predicted by Scherman and co-workers for a Q8·YL complex,^22^ but contrary to their prediction for a Q8·LY complex.^22^

**Fig. 5:**
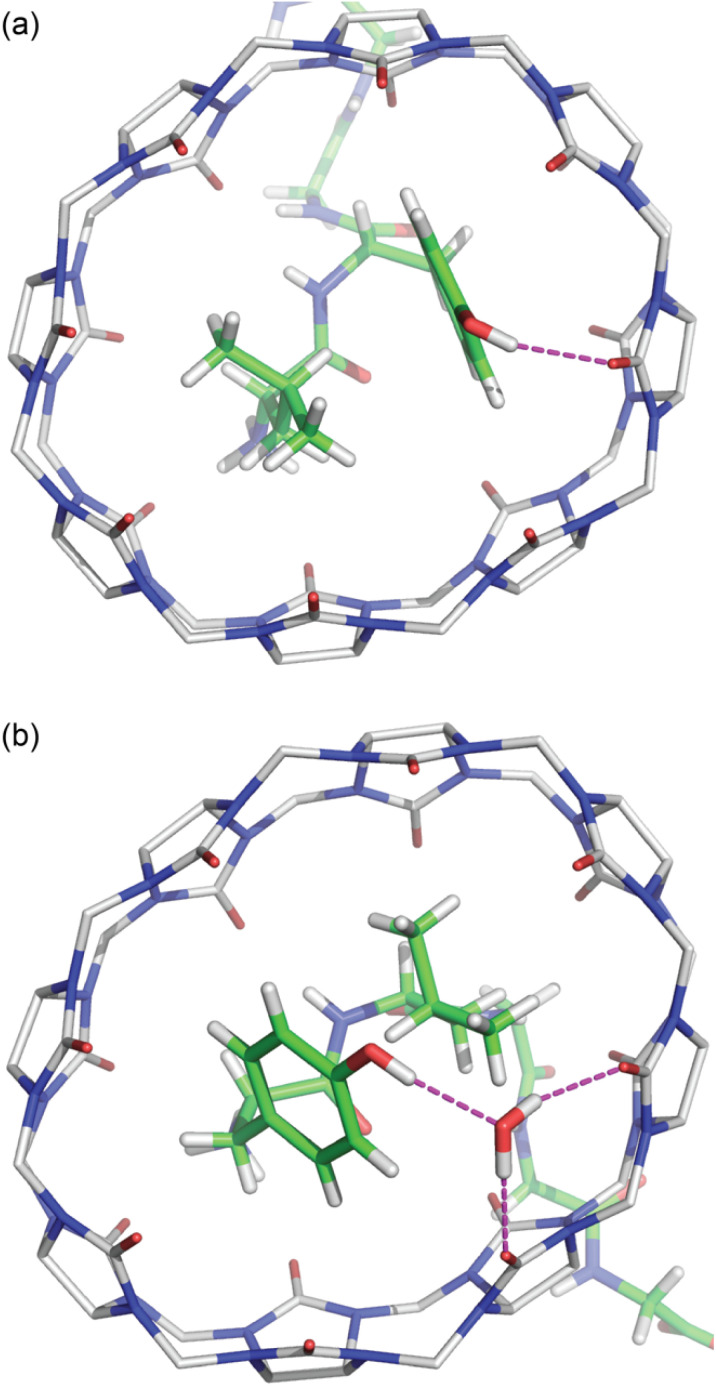
Bottom view of the crystal structures of (a) Q8·LYGGG and (b) Q8·YLGGG. Highlighted here are electrostatic interactions between the Tyr phenolic OH group and the Q8 portal. Host molecules have hydrogens omitted for clarity. The molecules are coloured as follows: Q8 carbons in grey, peptide carbons in green, nitrogens in blue, oxygens in red, and hydrogens in white.

The Tyr and Leu side chains in the Q8·YLGGG complex are bound more deeply within the Q8 cavity than they are in the Q8·LYGGG complex (Fig. 4). The positions of the Leu and Tyr α-carbons in the Q8·YLGGG structure are approximately 0.5 Å inside of the plane of Q8 carbonyl oxygens, whereas the Tyr α carbons in the Q8·LYGGG are aligned with the Q8 carbonyl oxygens. The side chains of the Tyr and Leu residues in the Q8·YLGGG structure are therefore pushed more deeply into the cavity than in the Q8·LYGGG structure. The geometric constraint forces the Tyr side chain of YLGGG at an angle (compared to the Q8·LYGGG structure) and positions the Tyr phenolic OH group outside of the plane of Q8 carbonyl oxygens and in the centre of the portal, such that a water molecule is needed to bridge them (Fig. 5). In the Q8·LYGGG structure, however, the Tyr phenolic OH group forms a direct hydrogen with a proximal C=O group on Q8.

**Fig. 6:**
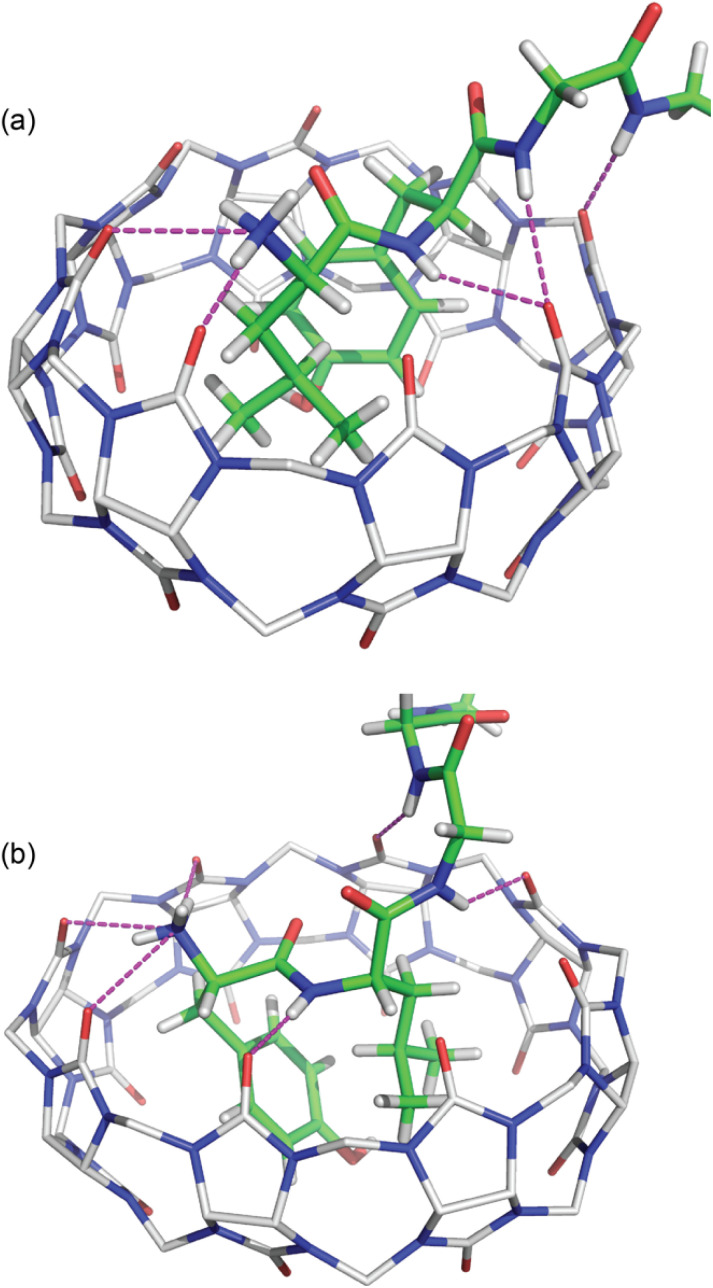
Crystal structures of (a) Q8·LYGGG and (b) Q8·YLGGG showing the hydrogen bonds as dashed lines between the peptide backbone and the Q8 portal. Q8 hydrogens have been omitted for clarity. The molecules are coloured as follows: Q8 carbons in grey, peptide carbons in green, nitrogens in blue, oxygens in red, and hydrogens in white.

In both structures there are several hydrogen bonds between the peptide backbone and the proximal carbonyl groups on Q8 (Fig. 6).‡‡We follow the definition of hydrogen bonding from the 2011 recommendations of the IUPAC.^66,67^ Briefly, in a D–H⋯A system, the bonding is an attractive interaction stemming from either electrostatics, dispersion, or charge transfer; the donor (D) and acceptor (A) are more electronegative than H; the DHA angle approaches 180° with a minimum of 110°; the H⋯A distance ranges from ∼1.0 to 2.6 Å, and where there is either spectroscopic or crystallographic evidence for bond formation.^66,67^ In the Q8·LYGGG structure, the Tyr NH forms a hydrogen bond with a proximal C=O oxygen on Q8, which also forms a bifurcated hydrogen bond with the Gly3 NH. The Gly4 NH forms a hydrogen bond with the neighbouring C=O group. In the Q8·YLGGG structure, however, the Tyr NH, Gly3 NH, and Gly4 NH each form a hydrogen bond with a unique, proximal C=O oxygen. The protonated N-terminal ammonium groups in both complexes form hydrogen bonds with proximal C=O groups on Q8. In the Q8·LYGGG structure, the Leu nitrogen is ≤3.0 Å from two carbonyl oxygens, whereas in the Q8·YLGGG structure, the Tyr nitrogen is ≤3.0 Å from three carbonyl oxygens. The C-terminal carboxylate groups in both complexes do not directly interact with the equator of neighbouring complexes, as observed in the Q8·GGLYGGG structure,^23^ but rather they occupy the spaces in between the complexes in the crystal and form hydrogen bonds with the interstitial water molecules. This observation is similar to the occupancy of sulfate, oxonium, and disordered water in the inter-macrocycle spaces in the crystal structure of unbound Q8.^63^

Although the crystal structures provide information about the binding behaviour of Q8 in the solid state, we believe they suggest a molecular basis for the extraordinary selectivity of Q8 for LYGGG *vs.* YLGGG observed in solution. Simultaneous inclusion of the Tyr and Leu side chains within the Q8 cavity presents significant steric demands, and Q8 is highly rigid with significant torsional strain. In the Q8·LYGGG complex, the host shows minimal puckering, whereas in the Q8·YLGGG complex, the significant deformation of Q8 indicates poor shape complementarity and an energetic cost for binding compared to the Q8·LYGGG complex. In both structures, the N-terminal ammonium group is positioned proximal to the portal, driven by highly stabilizing ion–dipole interactions. This position forces the side chain of the N-terminal residue to insert fully within the Q8 cavity. If the first residue is Tyr, then the large side chain is forced into a conformation in which the ring protrudes from the opposite portal, costing a direct hydrogen bond with C=O groups at that portal and possibly a significant amount of complex stability compared to the Q8·LYGGG complex. If the first residue is Leu, then the aliphatic side chain is able to be fully buried within the Q8 cavity. Therefore, we propose that the 1.2 nM affinity observed in the Q8·LYGGG complex is conferred by the optimal shape of the aliphatic residue at the first position and aromatic residue at the second position that enables inclusion within the Q8 cavity without significant distortion of the macrocycle and while forming a direct hydrogen bond between the Tyr OH group and a Q8 C=O group and maintaining numerous electrostatic interactions between the peptide backbone and the Q8 portal.

Although we were unable to obtain crystal structures for Q8 in complex with KFGGY or FKGGY, we believe that the subnanomolar affinity of Q8 for KFGGY and the 1000-fold selectivity *versus* FKGGY might be explained by factors similar to those observed in the Q8·LYGGG and Q8·YLGGG crystal structures. In both sets of peptides, the higher affinity complex has an aliphatic residue at the first position and an aromatic residue at the second position (*i.e.*, LY *vs.* YL and KF *vs.* FK). Pair inclusion of KF would be expected to induce significantly less puckering of Q8 than inclusion of FK, while allowing the positioning of both the N-terminal ammonium and Lys ε-ammonium proximal to Q8 carbonyl oxygens. Further structural studies would be needed to confirm this hypothesis.

## Conclusions

This study presents the unexpected observation of subnanomolar binding affinity between a synthetic receptor and an unmodified peptide in neutral aqueous buffer. This result is remarkable in light of the exceptionally small binding site, only two amino acid residues. Unlike other studies of the pair-inclusion motif, the results presented here demonstrate extraordinary selectivity of Q8 for the dipeptide binding sites Lys–Phe and Leu–Tyr *versus* their sequence isomers Phe–Lys and Tyr–Leu, respectively. Comparative analysis of the isomeric crystal structures of Q8·LYGGG and Q8·YLGGG reveals remarkable electrostatic and shape complementarity between host and guest that confers such stability and sequence selectivity. Given the many reports demonstrating successful incorporation of the tripeptide FGG affinity tag at the N-terminus of proteins for micromolar recognition by Q8, we anticipate that incorporation of KF or LY as N-terminal protein affinity tags should enable predictive and selective recognition by Q8 at subnanomolar concentrations. This capability would be an important step toward the development of applications such as protein sensing or inhibition under physiologically relevant conditions while having minimal impact on the structure and properties of the tagged protein. The future development of derivatization strategies for Q8 should greatly enhance the applicability of this approach to protein recognition.

## Data availability

All data including experimental and analytical details are in the ESI.†

## Author contributions

P. S., M. B. E., L. B. K., H. D. A., D. J. W., and A. R. U. conducted experiments and analytical studies. P. S., M. B. E., and A. R. U. wrote the manuscript.

## Conflicts of interest

There are no conflicts to declare.

## Supplementary Material

SC-015-D4SC01122H-s001

SC-015-D4SC01122H-s002
