# Supplementary material for: Peptide recognition by a synthetic receptor at subnanomolar concentrations
Source: Chem Sci. 2024 Mar 12;15(14):5133–42. doi: 10.1039/d4sc01122h (PMC10988627; doi:10.1039/d4sc01122h)
Supplement: SC-015-D4SC01122H-s001 [file SC-015-D4SC01122H-s001.pdf]

Electronic Supplementary Material (ESI) for Chemical Science.
This journal is © The Royal Society of Chemistry 2024

Supporting Information

Peptide Recognition by a Synthetic Receptor at Subnanomolar Concentrations

Paolo Suating,[1] Marc B. Ewe,[1] Lauren B. Kimberly,[1] Hadi D. Arman,[2] Daniel J. Wherritt,[2] and Adam R. Urbach[1*]

[1] Department of Chemistry, Trinity University, 1 Trinity Place, San Antonio, TX 78212 USA
[2] Department of Chemistry, University of Texas at San Antonio, 1 UTSA Circle, San Antonio TX 78249 USA

[*] To whom correspondence should be addressed: aurbach@trinity.edu

Pages	Contents
S2–S8	Experimental Details
S9-S10	Crystallographic Data
S11–S13	Isothermal Titration Calorimetry (ITC) Data
S14–S25	^{1}H Nuclear Magnetic Resonance (^{1}H NMR) Spectroscopy Data
S26-S29	Mass Spectrometry (MS) Data
S30	References

Experimental Details

Materials. Commercially available compounds were of at least analytical purity grade and were used with further purification unless otherwise specified. Anhydrous dichloromethane (DCM), anhydrous diethyl ether, methyl viologen dichloride hydrate (MV), iodomethane, (ferrocenylmethyl)dimethylamine ($FcCH_2NMe_2$), and AmberChrom 1X4 chloride form resin were purchased from Millipore–Sigma. HPLC-grade water, HPLC-grade acetonitrile (MeCN), Phenomenex aluminium-backed silica TLC plates (60 Å, F254), monosodium phosphate, and disodium phosphate were purchased from VWR. Ultra-pure water (18 MΩ·cm, 2 ppb TOC) was obtained from an in-house Elga Ultra water purification system. Cucurbit[8]uril (Q8) was synthesized using previously published methods,[1] or purchased from Strem Chemicals. Deuterium oxide (99.9%) was purchased from Cambridge Isotope Laboratories. The following sequences were purchased from Genscript Biotech: peptides H-KFGGY-OH, H-FKGGY-OH, H-LYGGG-OH, and H-YLGGG-OH, and all peptides had unprotected *N*-termini and *C*-termini.

Sodium phosphate buffer (10 mM, pH 7.0) was prepared by dissolving NaH_2PO_4 (6.13 mmol) and Na_2HPO_4 (3.87 mmol) in ultra-pure water (1.00 L), and then adjusting the pH using either HCl (12.1 M) or NaOH (50% w/w in H_2O). The concentration of tyrosine-containing peptides was determined using UV spectroscopy (ε_{275} = 1420 M^{-1} cm^{-1}). The concentration of Q8 was determined via calorimetric titration using a solution of MV that had been standardized by UV spectroscopy (ε_{257} = 20400 M^{-1} cm^{-1}).

Electrospray Ionization Time-of-Flight Mass Spectrometry. Mass spectra of purified peptides and their complexes with Q8 (Figures S18-25) were acquired by infusion using an Agilent 6230 TOF LC/MS mass spectrometer with an electrospray ion source in the positive ion mode. Samples

were dissolved in pure water at a concentration of 100 μM purified peptide in the absence and presence of one molar equivalent of Q8.

Isothermal Titration Calorimetry (ITC). Titrations were carried out in 10 mM pH 7.0 phosphate-buffered H_2O at 300 K using either a VP-ITC calorimeter (Malvern, Inc.), or a nanoITC LV (low volume) calorimeter (TA Instruments) (Figure S1-S8). In a typical experiment, Q8 was in the sample cell at concentrations in the range of 30–50 μM.

VP-ITC: (Figure S5) The peptide solution was in the syringe in the concentration range 300–500 μM. The titration schedule consisted of 28 consecutive injections of 10 μL with at least a 200 s interval between injections. Heats of dilution, measured by titrating beyond saturation, were subtracted from each dataset. All solutions were degassed prior to titration. The data were analysed using Origin software and fit by non-linear regression to the binary equilibrium binding model (non-interacting sites) supplied with the software to determine molar enthalpy, equilibrium association constant, and binding stoichiometry. These values were used to calculate the free energies of binding and the entropic contributions to the binding free energies.

nanoITC LV: (Figures S1, S3, S6, S8) The peptide solution was in the syringe at the concentration range 198–330 μM. The titration schedule consisted of 28 successive injections of 1.76 μL (41 injections of 1.2 μL in the case of H-YLA-NH_2) and with at least 150 s between injections. Heats of dilution, measured by titrating beyond saturation, were subtracted from each dataset. All solutions were degassed prior to titration. The data were analysed using the proprietary nanoAnalyze software and fit by non-linear regression to the independent equilibrium binding model supplied with the software to determine molar enthalpy, equilibrium association constant,

and binding stoichiometry. These values were used to calculate the free energies of binding and the entropic contributions to the binding free energies.

Competitive ITC titrations to determine affinities >10^7 M^{-1} were carried out on the nanoITC LV as above, except for the presence of 100 equiv. of a weak competitive guest (G^{weak}) in the cell along with Q8 (Figures S2, S4, S7). This titration yields ΔH^{obs}, and K_a^{obs} for the displacement. The binding constant of the weak competitor in the cell (K_a^{weak}) was previously determined by direct titration. The binding constant of the high-affinity guest (K_a^{strong}) was then determined by the the following equation:

$$K_a^{strong} = K_a^{obs} K_a^{weak} [G^{weak}].$$

Experimental conditions that yielded K_a^{obs} also gave observed molar binding enthalpies ΔH^{obs}. This binding enthalpy is the sum of the binding enthalpy of the weak competitor (ΔH^{weak}) and of the strong guest (ΔH^{strong}) and is based on the thermodynamic cycle of the form:

$$\begin{array}{rcll} Q8 + G^{weak} & \rightleftharpoons & Q8\bullet G^{weak} & \Delta H^{weak} \\ Q8\bullet G^{weak} + G^{strong} & \rightleftharpoons & Q8\bullet G^{strong} + G^{weak} & \Delta H^{obs} \\ \hline Q8 + G^{strong} & \rightleftharpoons & Q8\bullet G^{strong} & \Delta H^{strong} \end{array}$$

Thus, the molar enthalpy of binding of the strong guest is given by

$$\Delta H^{weak} + \Delta H^{obs} = \Delta H^{strong}.$$ [2]

Nuclear Magnetic Resonance (NMR) Spectroscopy: (Figures 2, S9-S17) All 1-D and 2-D NMR spectra were collected in D_2O (δ 4.790) at 21 °C using either a Varian 500 MHz instrument with an operating frequency of 499.6 MHz, or a Bruker Avance NEO 500 MHz instrument with an operating frequency of 500.13 MHz. NMR spectral data were processed using MNova 14 (Mestrelab Research SL). Multiplicity abbreviations are as follows: s – singlet; t – triplet. Signal

presaturation of residual protiated solvent was used as necessary. To aid in solubility, the peptides and their 1:1 complexes with Q8 were dissolved in 10 mM sodium phosphate-buffered D_2O, $pH_{apparent}$ 7.1,[3] which was prepared as follows: NaH_2PO_4 (5.54 mmol) and Na_2HPO_4 (3.49 mmol) were dissolved in D_2O (100 g, 90.334 mL) to yield a solution of 100 mM pH 7.0 ($pH_{apparent}$ 7.1) sodium phosphate-buffered D_2O, which was diluted to 10 mM prior to use.

Correlation spectroscopy (COSY) spectra were acquired with a 2 s relaxation delay and with a spectral width of 8012 Hz. The COSY spectrum of the 1:1 Q8•KFGGY complex was recorded with 1024t1 * 1024t2 complex points. The 2-D nuclear Overhauser effect spectrum of free KFGGY and the rotating-frame Overhauser effect spectrum of the 1:1 Q8•KFGGY were acquired using a 2 s relaxation delay, a 500 ms mixing (spin-lock) time (250 ms for the free peptide), a spectral width of 7500 Hz, and was recorded with 1024t1 * 512t2 complex points. Chemical shift values were referenced relative to TSP via the residual solvent resonance at 4.77 ppm.

Competition Experiments Using ^1H NMR spectroscopy. The competition experiment to determine the binding affinity of H-KFGGY-OH to Q8 was performed according to the Isaacs competition method in order to provide a benchmark for the competitive ITC titration experiments (Figure S14).[4] Briefly, to a mixture of known concentrations of (ferrocenylmethyl)trimethylammonium chloride ($FcNMe_3$) and H-KFGGY-OH in 10 mM sodium phosphate-buffered D_2O, $pH_{apparent}$ 7.1, was added a limiting molar quantity of Q8. The mixture was subjected briefly to 1-3 cycles of sonication and heating at 60 °C to fully solubilize the Q8. NMR spectra were acquired with a relaxation delay of 30 s to improve the accuracy of signal integration. The total concentration of peptide in the mixture ($[peptide]_o$) was determined by UV

spectroscopy (ε_{275} = 1420 M^{-1} cm^{-1}). Therefore, the total integration of the aromatic signals of peptide was used as the benchmark for determining the concentrations of other species in the mixture via relative integration. The total concentration of Q8 ($[Q8]_o$) in the mixture was determined using the following equation:

$$[Q8]_o = \frac{I_{Q8}}{I_{Ar}} \times \frac{N_{Ar}}{N_{Q8}} \times [\text{peptide}]_o$$

where I_{Q8} is the integration value of a Q8 signal; N_{Q8} is the number of hydrogen atoms corresponding to that Q8 signal (i.e., 16); I_{Ar} is the total integration value of all aromatic signals; and N_{Ar} is the total number of hydrogen atoms corresponding to all signals in the aromatic region (i.e., 9). The same method was used to find the equilibrium concentrations of unbound peptide ($[\text{peptide}]_{eq}$) and bound peptide ($[\text{peptide}\cdot Q8]_{eq}$). Using the conservation of mass, the equilibrium concentration of bound $FcNMe_3$ ($[FcNMe_3\cdot Q8]_{eq}$) is given by

$$[FcNMe_3\cdot Q8]_{eq} = [Q8]_o - [\text{peptide}\cdot Q8]_{eq}$$

Similarly, the equilibrium concentration of free $FcNMe_3$ ($[FcNMe_3]_{eq}$) can be found by mass balance

$$[FcNMe_3]_{eq} = [FcNMe_3]_0 - [FcNMe_3\cdot Q8]_{eq}$$

These values can be used to determine the equilibrium constant for the competition reaction

$$FcNMe_3 \bullet Q8 + \text{peptide} \rightleftharpoons \text{peptide} \bullet Q8 + FcNMe_3$$

which is represented by the following equilibrium expression and is also the ratio of the equilibrium dissociation constant (K_d) values for the $FcNMe_3$•Q8 and peptide•Q8 complexes.

$$K_{comp} = \frac{[\text{peptide}\cdot Q8]_{eq}[FcNMe_3]_{eq}}{[FcNMe_3\cdot Q8]_{eq}[\text{peptide}]_{eq}} = \frac{K_{d\,(FcNMe_3\bullet Q8)}}{K_{d\,(\text{peptide}\bullet Q8)}}$$

Therefore, the K_d value for the peptide•Q8 complex was determined as

$$K_{d\ (peptide\bullet Q8)} = \frac{K_{d\ (FcNMe_3\bullet Q8)}}{K_{comp}}$$

Synthesis of (ferrocenylmethyl)trimethylammonium chloride ($FcNMe_3$). To a round-bottomed flask fitted with a magnetic stir bar and flushed with nitrogen gas was added (ferrocenylmethyl)dimethylamine (1.0 mmol, 0.198 mL) and anhydrous MeCN (5.0 mL). Iodomethane (2.5 mmol, 0.156 mL) was then added in one portion, and the mixture was allowed to stir at room temperature for 90 min. Another portion of iodomethane was added (2.5 mmol, 0.156 mL), and the mixture was allowed to stir for an additional 90 min. Thin-layer chromatography was used to monitor the reaction (R_f = 0.38, 10:1 DCM/MeOH). Anhydrous diethyl ether (20 mL) was then added to the mixture to induce precipitation of the iodide salt of the product. The suspension was then filtered, and the filter cake was washed with additional diethyl ether until the filtrate was colourless. The solids were dried under high vacuum for 30 min to yield (ferrocenylmethyl)trimethylammonium iodide as an orange powder. The solids were then dissolved in ultra-pure H_2O (1 mL), passed through a short column loaded with AmberChrom 1X4 chloride form ion-exchange resin, and eluted using ultra-pure water until the eluate was colourless. The eluted solution was then flash-frozen in liquid nitrogen and lyophilized to dryness to yield (ferrocenylmethyl)trimethylammonium chloride as a deep yellow powder (0.265 g, 0.902 mmol, 90%). Spectroscopic data agree well with the literature.[5] ^{1}H NMR (D_2O, δ 4.79): δ 2.96 (s, 9H), 4.28 (s, 5H), 4.40 (s, 2H), 4.44 (t, J = 1.9 Hz, 2H), 4.52 (t, J = 1.9 Hz, 2H). ESI-TOF MS: m/z: $[M]^+$ Calcd for $C_{14}H_{20}FeN$ 256.098, Found 256.096; $[2M + Cl]^+$ Calcd for $C_{28}H_{40}ClFe_2N_2$ 549.162, Found 549.164.

X-ray Crystallography. Single crystals of the Q8•H-YLGGG-OH complex suitable for X-ray crystallography were grown by mixing H-YLGGG-OH (535 µL, 3.736 mM in 10 mM sodium phosphate-buffered D_2O, pH* 7.1) and dry Q8 (1.0 equiv., 3.943 mg) in a scintillation vial. Single crystals of the Q8•H-LYGGG-OH complex suitable for X-ray diffractometry were grown by mixing H-LYGGG-OH (355 µL, 3.45 mM in 10 mM sodium phosphate-buffered H_2O, pH 7.0) and dry Q8 (0.9 equiv., 2.2 mg) in a scintillation vial. To each suspension was added 10 mM sodium phosphate-buffered D_2O, $pH_{apparent}$ 7.1, to a final complex concentration of 1.0 mM. Each suspension was subjected to two cycles of sonication and heating to 70 °C for 20 minutes and cooling to room temperature, during which time plate-like crystals formed that were coloured under linearly polarized light.

Reflection data were collected at 100 K on a XtaLAB Synergy, Dualflex HyPix four-circle diffractometer using CuK_α radiation (λ = 1.54184 Å). All data were integrated with CrysAlisPro[6] and corrected for absorption using ABSPACK.[6] The structures were solved by dual methods with SHELXT and refined by full-matrix least-squares methods against F^2 using SHELXL.[7, 8] All non-hydrogen atoms were refined with anisotropic displacement parameters. Most of the hydrogen atoms of the investigated structure were located from difference Fourier maps. Finally, their positions were placed in geometrically calculated positions and refined using a riding model including those bound to heteroatoms. When the hydrogen atom location was not obvious from difference maps hydrogen atoms were placed in logical hydrogen bonding geometries. Isotropic thermal parameters of the placed hydrogen atoms were fixed to 1.2 times the U value of the atoms they are linked to (1.5 times for methyl groups, NH groups, OH groups, and H_2O). For YLGGG, a solvent mask was calculated and 164 electrons were found in a volume of 440 Å^3 in 1 void per

unit cell. This is consistent with the presence of 8 water molecules per formula unit which account for 160 electrons per unit cell. The structures were also treated as a two-component inversion twin resulting in a BASF of 0.14(10). For LYGGG, a solvent mask was calculated and 476 electrons were found in a volume of 1278 Å^3 in 1 void per unit cell. This is consistent with the presence of 44 water molecules per formula unit which account for 440 electrons per unit cell. The structures were also treated as a two-component inversion twin resulting in a BASF of 0.14(10). Calculations and refinement of the structure was carried out using Olex2 software.[9] The data were deposited to the Cambridge Crystallographic Data Center (deposition numbers 2312293 and 2314758) (Tables S1 and S2).

Table S1. Crystal Data and Structure Refinement for Q8•YLGGG

CCDC number	2313004
Empirical formula	$C_{69}H_{132}N_{37}O_{50}$
Formula weight	2280.11
Temperature [K]	100.01(10)
Crystal system	Monoclinic
Space group (number)	$P2_1$ (4)
a [Å]	13.11250(10)
b [Å]	21.09440(10)
c [Å]	18.85710(10)
α [°]	90
β [°]	107.2330(10)
γ [°]	90
Volume [Å^3]	4981.73(6)
Z	2
ρ_{calc} [gcm^{-3}]	1.520
μ [mm^{-1}]	1.120
F(000)	2410.0
Crystal size [mm^3]	0.24×0.23×0.1
Crystal color	clear colorless
Crystal shape	plate
Radiation	CuK_α (λ=1.54184 Å)
2θ range [°]	4.91 to 153.91 (0.79 Å)
Index ranges	$-16 \le h \le 14$<br>$-25 \le k \le 25$<br>$-22 \le l \le 23$
Reflections collected	112702
Independent reflections	20089<br>R_{int} = 0.0504<br>R_{sigma} = 0.0315
Completeness to θ = 67.684°	100 %
Data / Restraints / Parameters	20089 / 2 / 1401
Goodness-of-fit on F^2	1.031
Final R indexes [$I \geq 2\sigma(I)$]	R_1 = 0.0377<br>wR_2 = 0.0973
Final R indexes [all data]	R_1 = 0.0391<br>wR_2 = 0.0986
Largest peak/hole [eÅ^{-3}]	0.77/−0.28

Table S2. Crystal Data and Structure Refinement for Q8•LYGGG

CCDC number	2314758
Empirical formula	$C_{276}H_{381.4}N_{148}O_{132.2}$
Formula weight	7887.87
Temperature [K]	99.99(10)
Crystal system	Monoclinic
Space group (number)	C_2 (5)
a [Å]	34.6961(3)
b [Å]	14.63880(10)
c [Å]	18.86300(10)
α [°]	90
β [°]	90.7510(10)
γ [°]	90
Volume [Å^3]	9579.87(12)
Z	1
ρ_{calc} [gcm^{-3}]	1.367
μ [mm^{-1}]	0.948
F(000)	4131.0
Crystal size [mm^3]	0.12×0.08×0.04
Crystal color	clear colorless
Crystal shape	plank
Radiation	CuK_α (λ=1.54184 Å)
2θ range [°]	8.04 to 140.0 (0.83 Å)
Index ranges	$-42 \leq h \leq 42$<br>$-17 \leq k \leq 17$<br>$-22 \leq l \leq 19$
Reflections collected	88713
Independent reflections	17957<br>R_{int} = 0.0443<br>R_{sigma} = 0.0300
Completeness to θ = 67.7°	99.8 %
Data / Restraints / Parameters	17957 / 14 / 1317
Goodness-of-fit on F^2	1.069
Final *R* indexes [$I \geq 2\sigma(I)$]	R_1 = 0.0560<br>wR_2 = 0.1308
Final *R* indexes [all data]	R_1 = 0.0590<br>wR_2 = 0.1324
Largest peak/hole [eÅ^{-3}]	0.70/−0.34

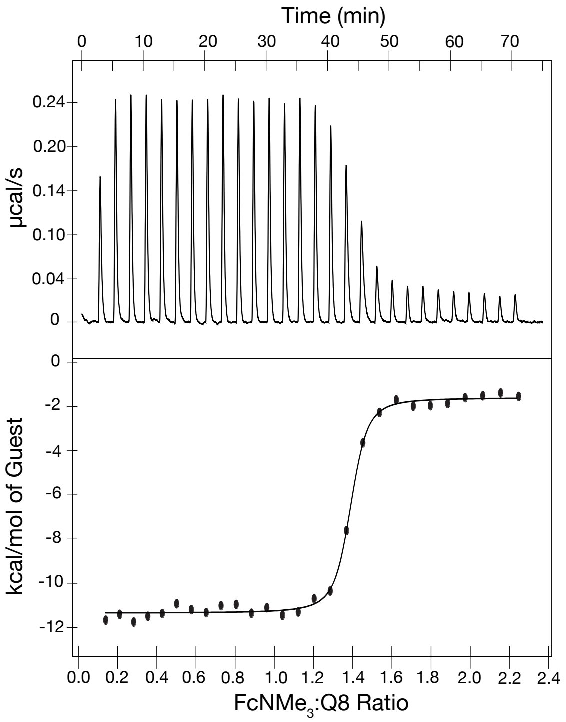

Figure S1: Representative isothermal titration calorigram of $FcNMe_3^+$ titrated into Q8.

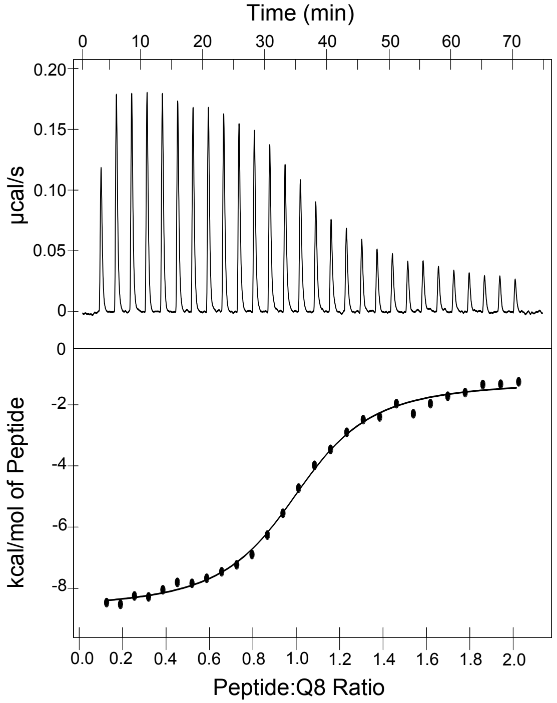

Figure S2: Representative isothermal titration calorigram of $FcNMe_3^+$ titrated into Q8 in the presence of 100 equiv. MV.

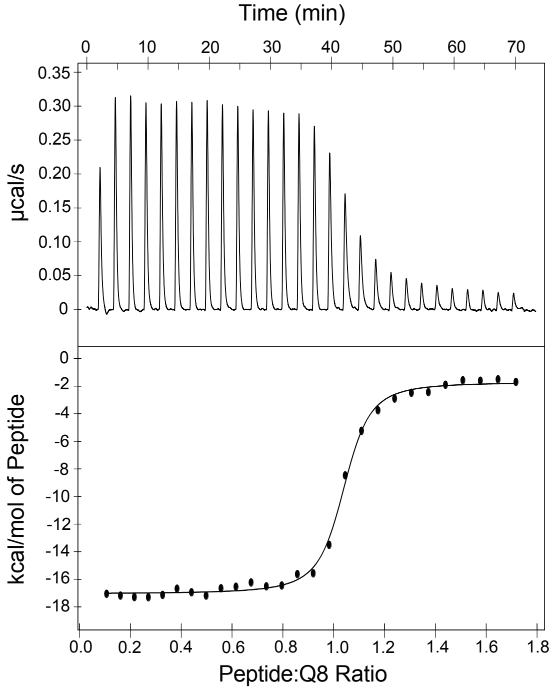

Figure S3: Representative isothermal titration calorigram of H-KFGGY-OH titrated into Q8.

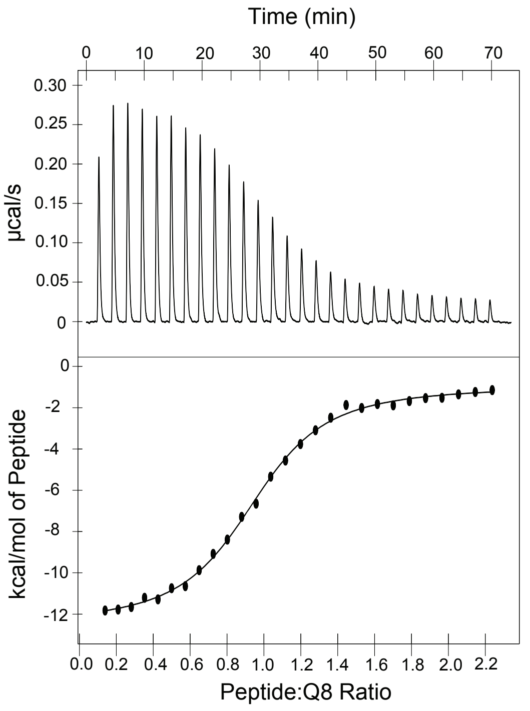

Figure S4: Representative isothermal titration calorigram of H-KFGGY-OH titrated into Q8 in the presence of 100 equiv. MV.

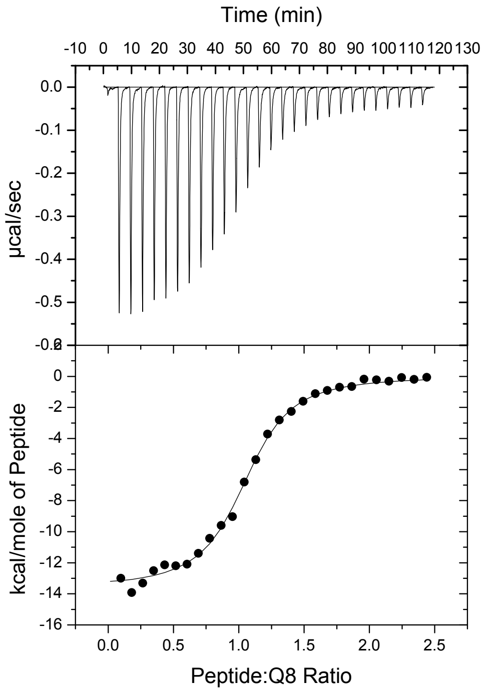

Figure S5: Representative isothermal titration calorigram of H-FKGGY-OH titrated into Q8.

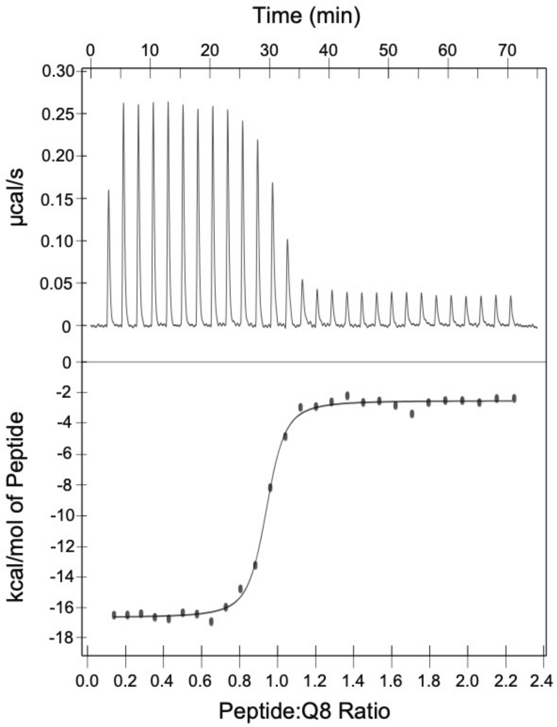

Figure S6: Representative isothermal titration calorigram of H-LYGGG-OH titrated into Q8.

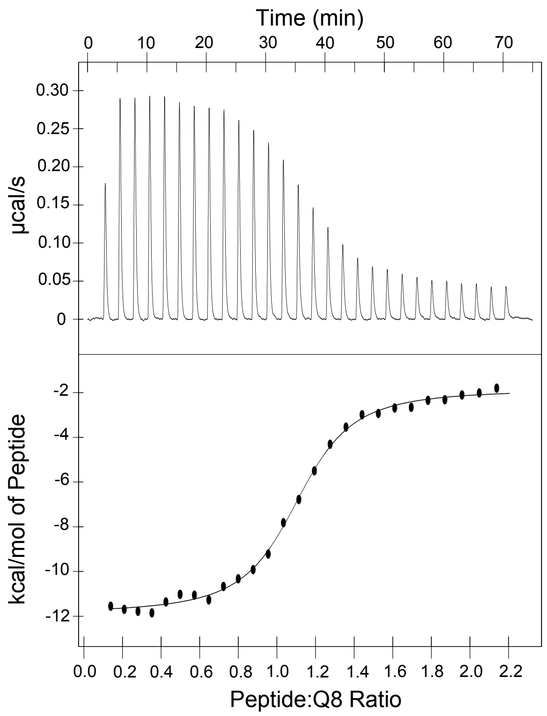

Figure S7: Representative isothermal titration calorigram of H-LYGGG-OH titrated into Q8 in the presence of 100 equiv. of MV.

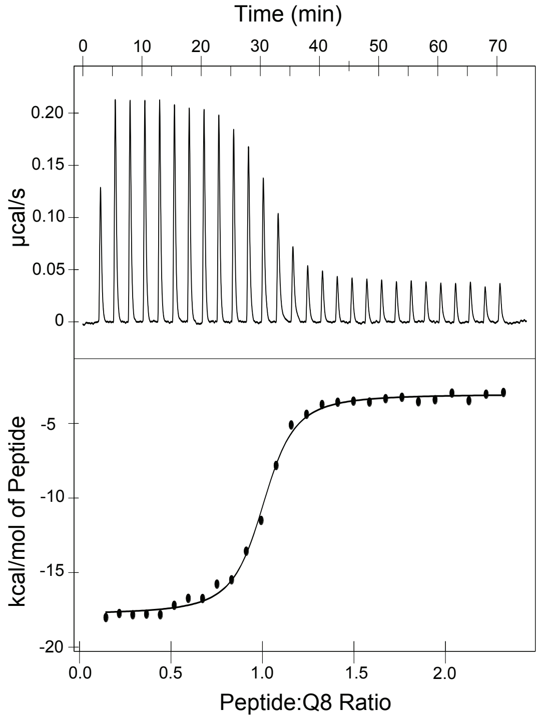

Figure S8: Representative isothermal titration calorigram of H-YLGGG-OH titrated into Q8.

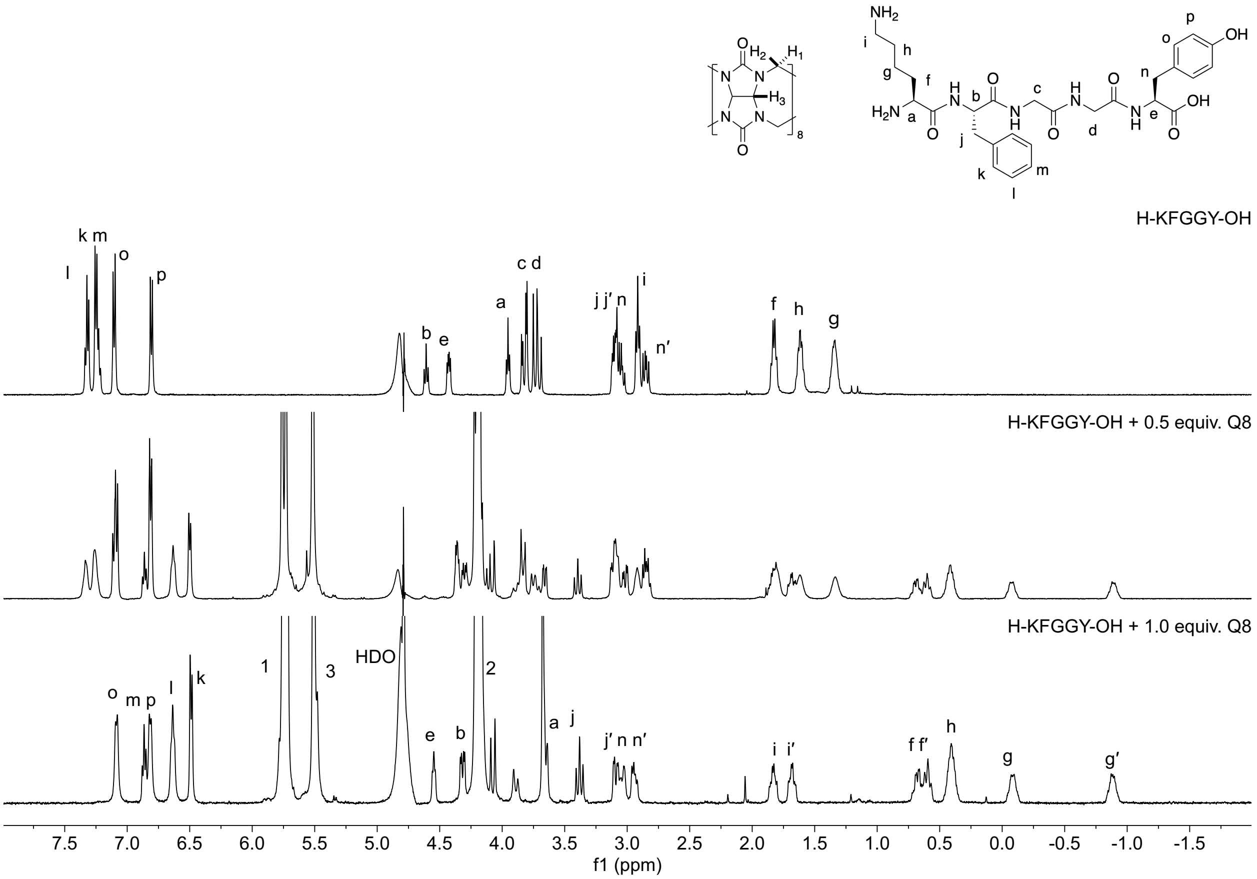

Figure S9: Stacked ^{1}H NMR spectra (D_2O, 500 MHz, 25 °C) of H-KFGGY-OH with (top–bottom) 0 equiv. Q8, 0.5 equiv. Q8, and 1.0 equiv. Q8.

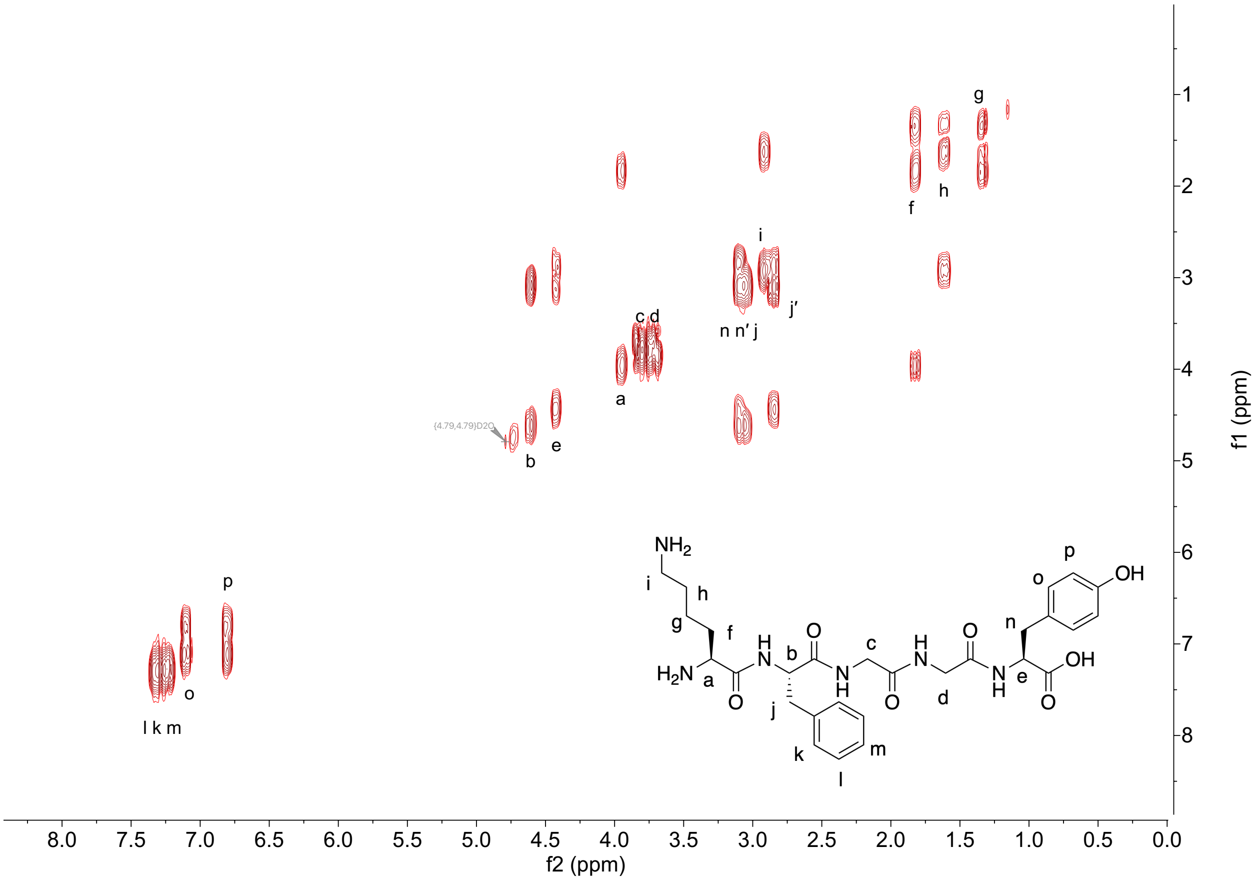

Figure S10: ${}^{1}H$-${}^{1}H$ COSY spectrum (D_2O, 500 MHz, 25 °C) of H-KFGGY-OH.

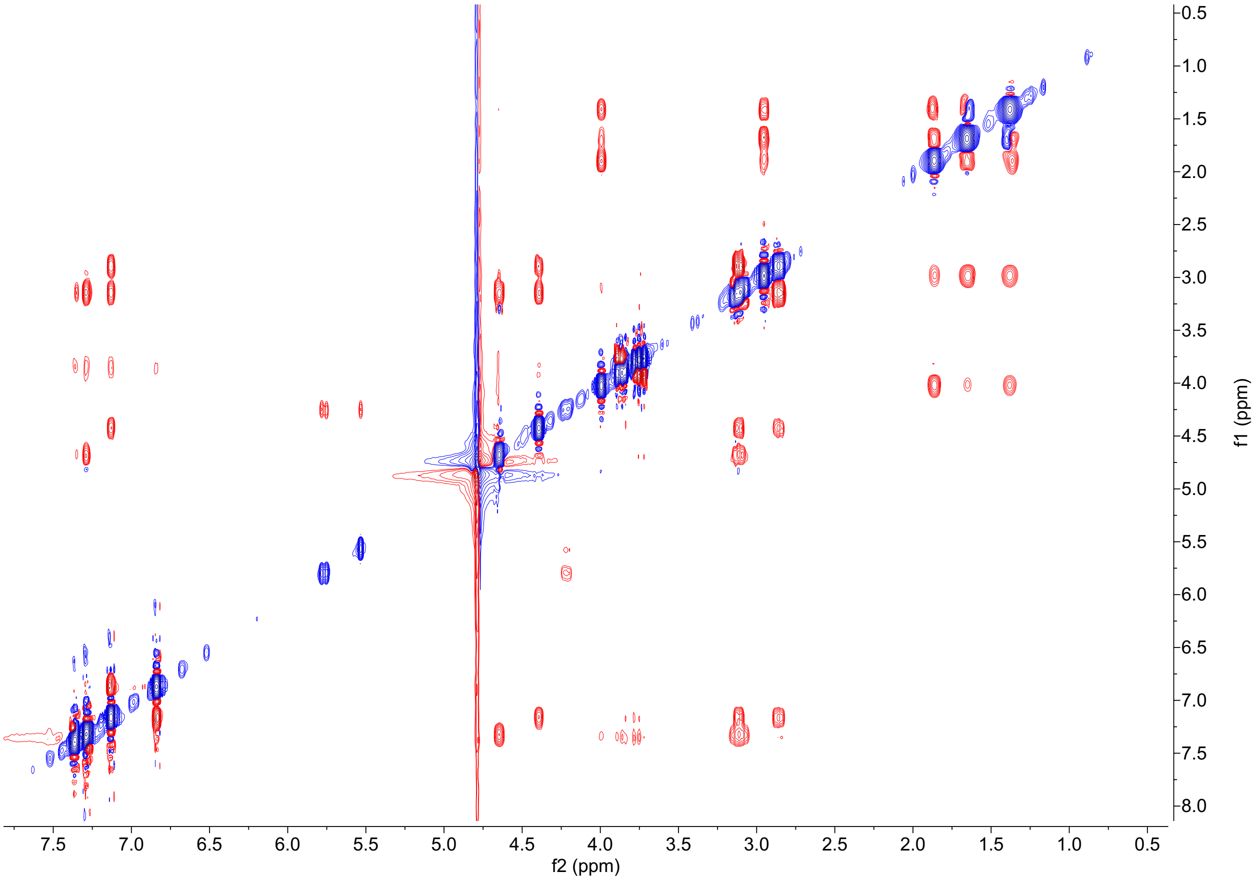

Figure S11: Full 2-D NOESY spectrum (D_2O, 500 MHz, 25 °C, mixing time 200 ms) of H-KFGGY-OH.

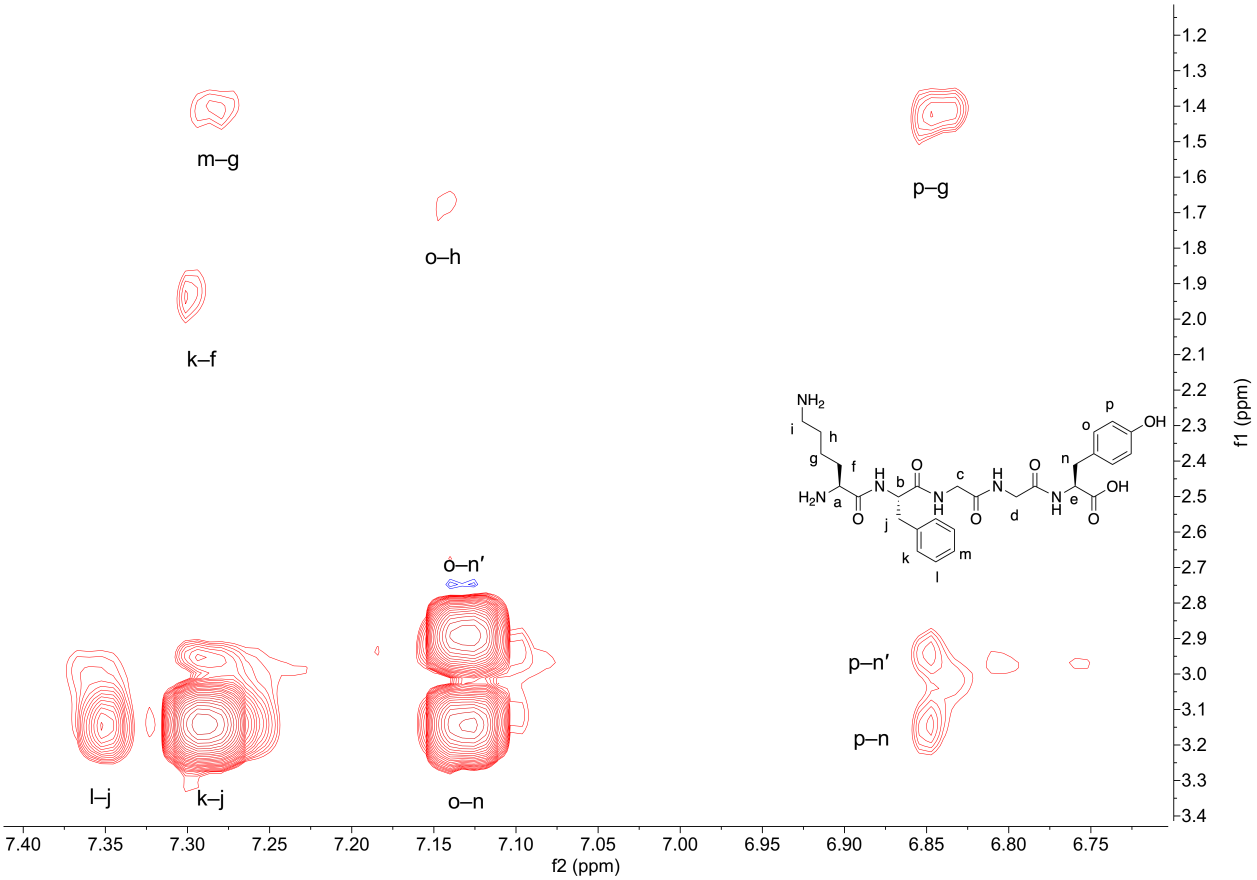

Figure S12: Partial 2-D NOESY spectrum (D_2O, 500 MHz, 25 °C, mixing time 200 ms) of H-KFGGY-OH. The image shows the zoomed in view of the full spectrum highlighting the NOEs between the Lys, Phe, and Tyr side chains. Peak intensities have been increased for this view.

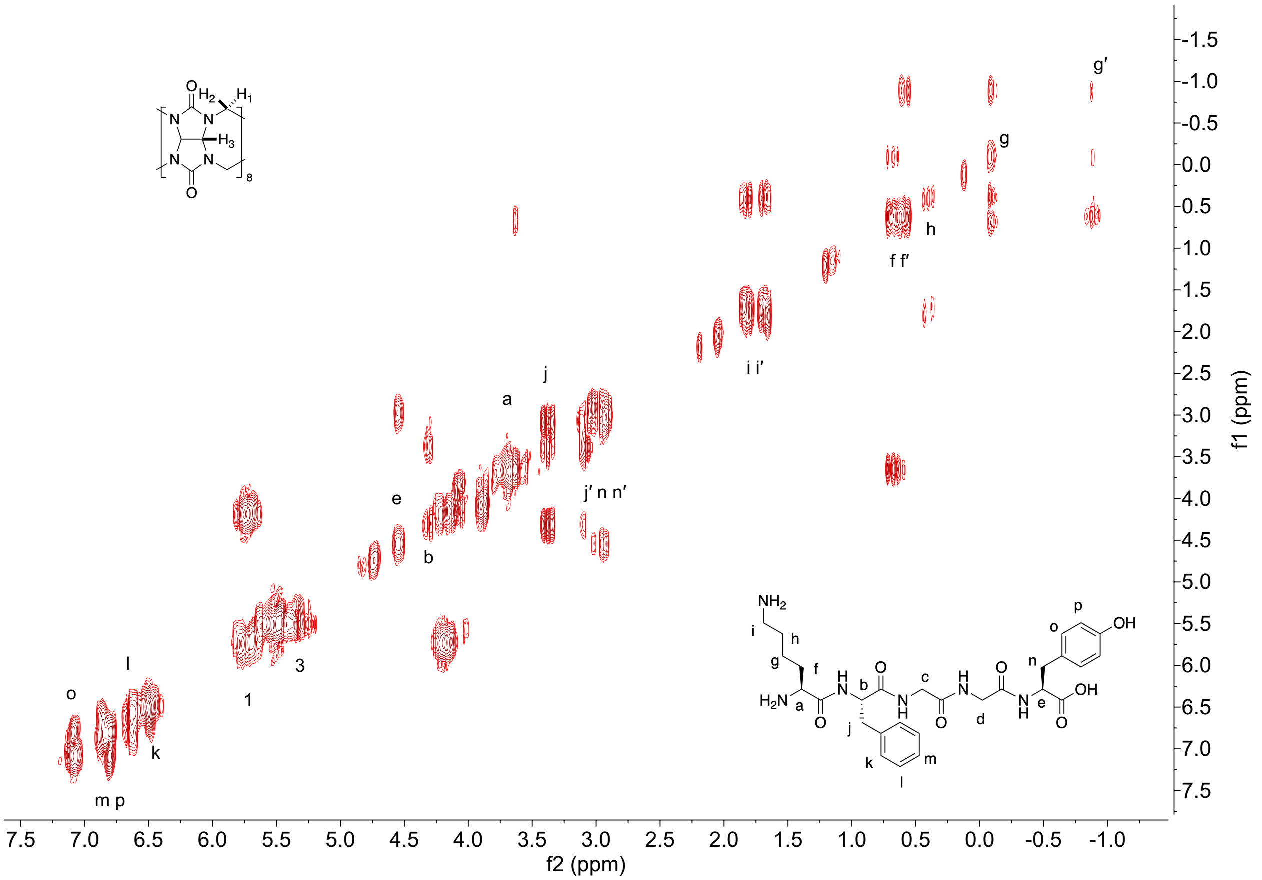

Figure S13: ^{1}H-^{1}H COSY spectrum (D_2O, 500 MHz, 25 °C) of an equimolar mixture of H-KFGGY-OH and Q8.

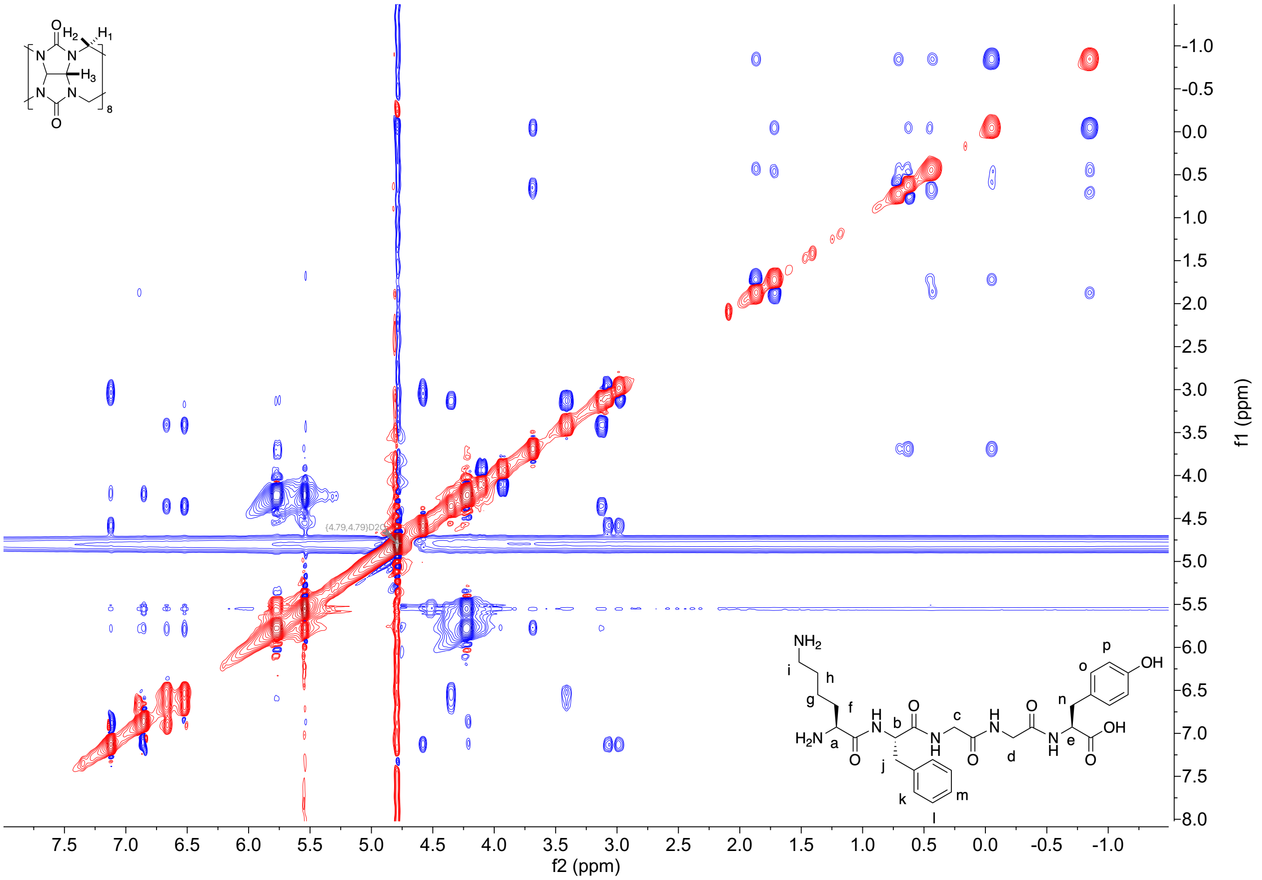

Figure S14: Full 2-D ROESY spectrum (D_2O, 500 MHz, 25 °C, mixing time 500 ms) of an equimolar mixture of H-KFGGY-OH and Q8.

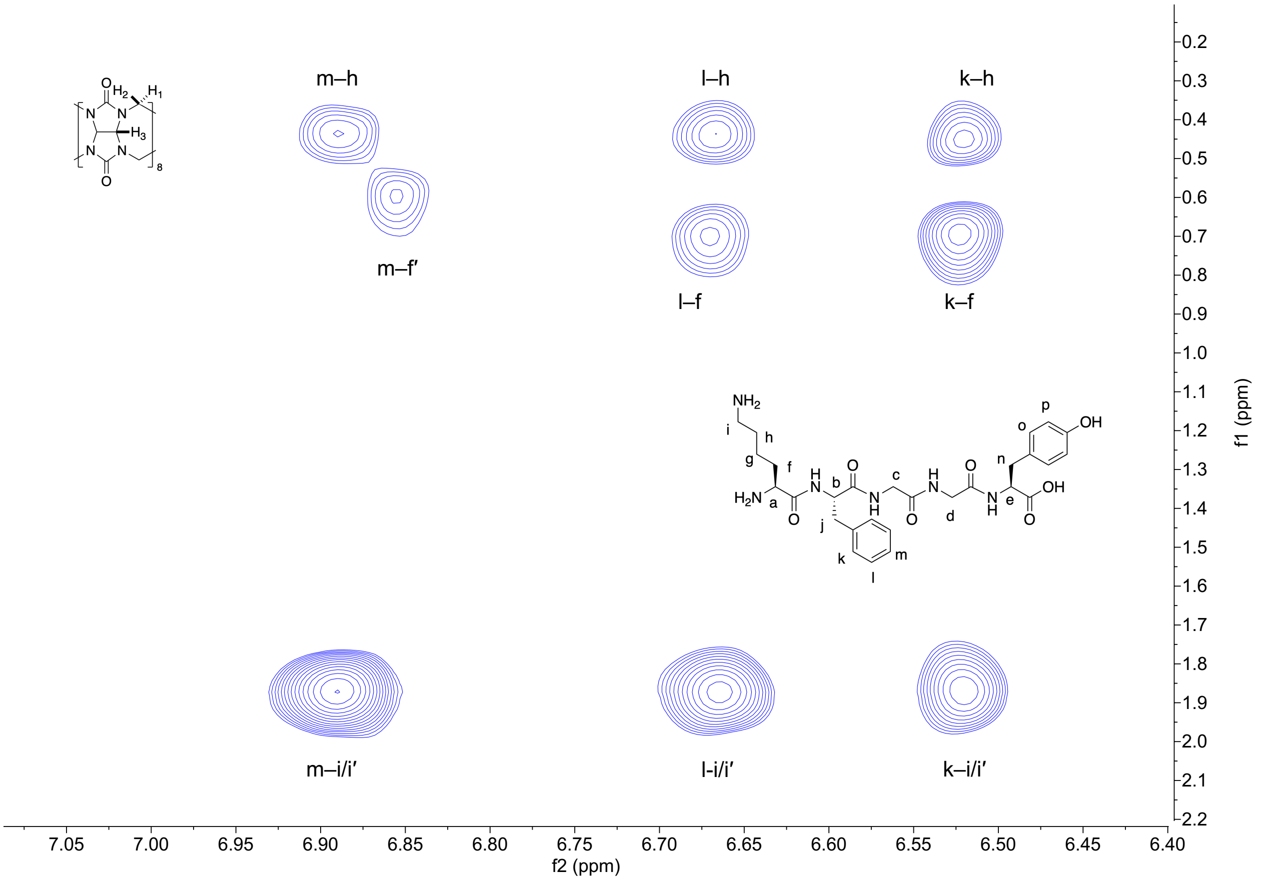

Figure S15: Partial 2-D ROESY spectrum (D_2O, 500 MHz, 25 °C, mixing time 500 ms) of an equimolar mixture of H-KFGGY-OH and Q8. The image shows the zoomed in view of the full spectrum highlighting NOEs between the Lys and Phe side chains. Peak intensities have been increased for this view.

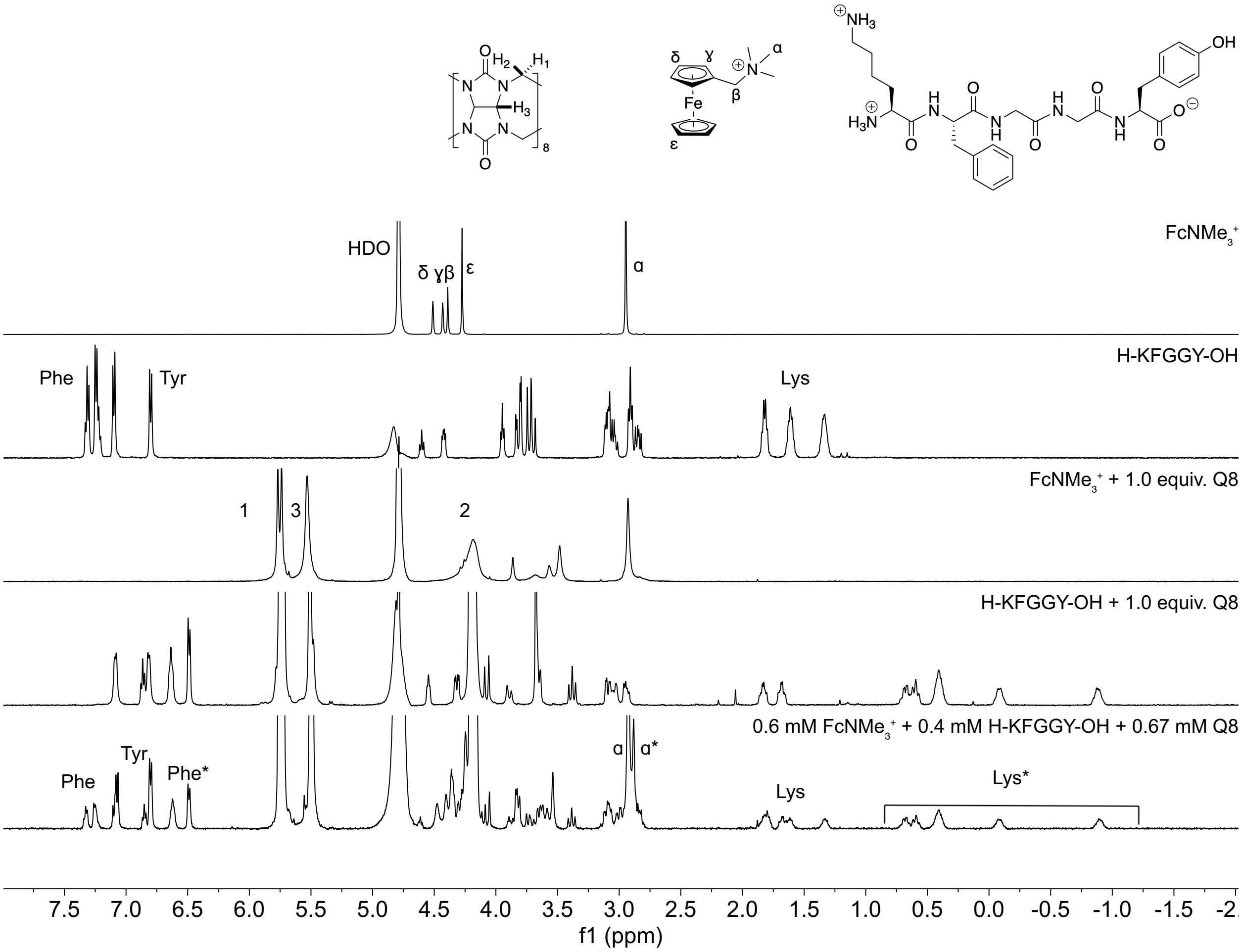

Figure S16: Stacked ^{1}H NMR spectra (D_2O, 500 MHz, 25 °C) of (*top–bottom*): (ferrocenylmethyl)trimethylammonium, $FcNMe_3^+$; H-KFGGY-OH; $FcNMe_3^+$ with 1.0 equiv. Q8; H-KFGGY-OH with 1.0 equiv. Q8; representative competition mixture of a 3:2 molar ratio of $FcNMe_3^+$ /H-KFGGY-OH with a limiting quantity of Q8. Signals marked with an asterisk denote protons bound inside Q8.

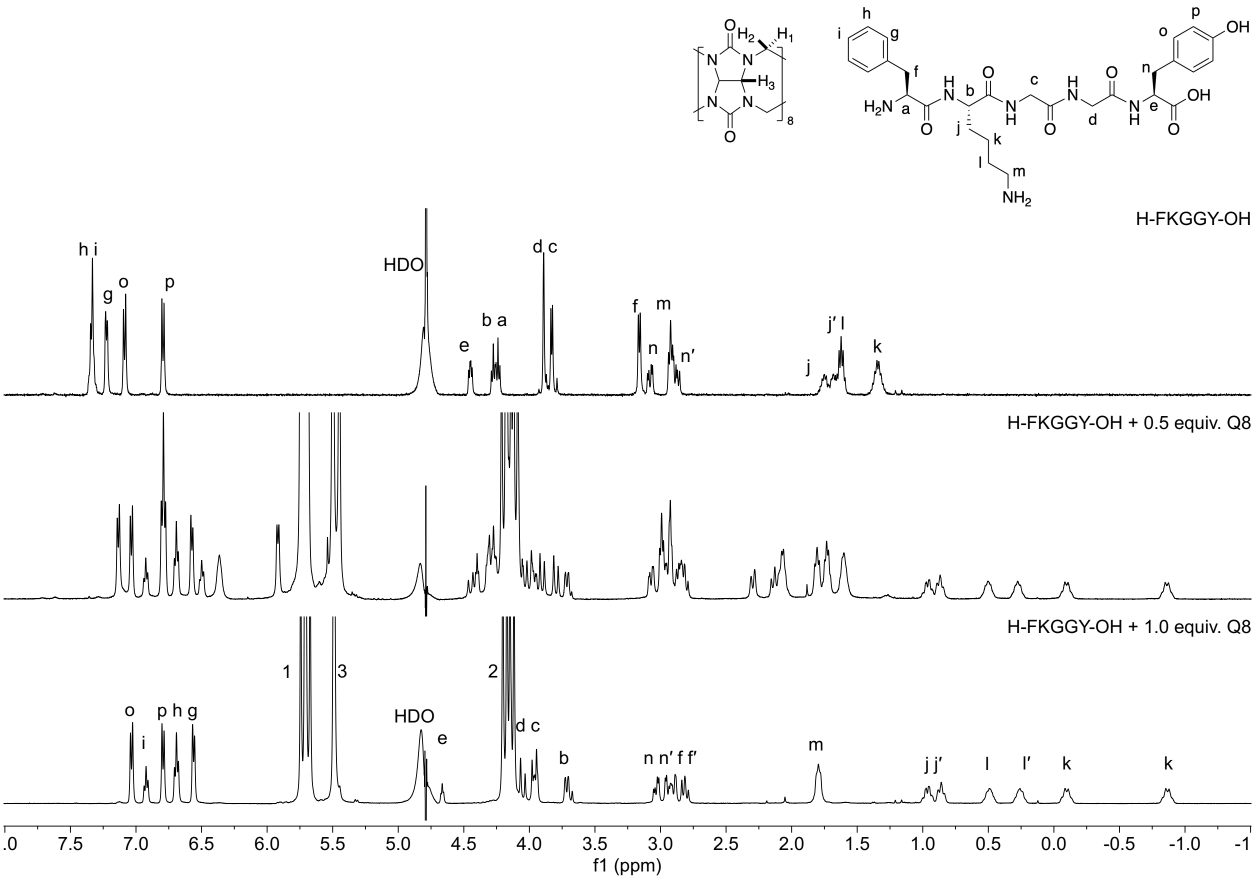

Figure S17: Stacked ^{1}H NMR spectra (D_2O, 500 MHz, 25 °C) of H-FKGGY-OH with (top–bottom) 0 equiv. Q8, 0.5 equiv. Q8, and 1.0 equiv. Q8.

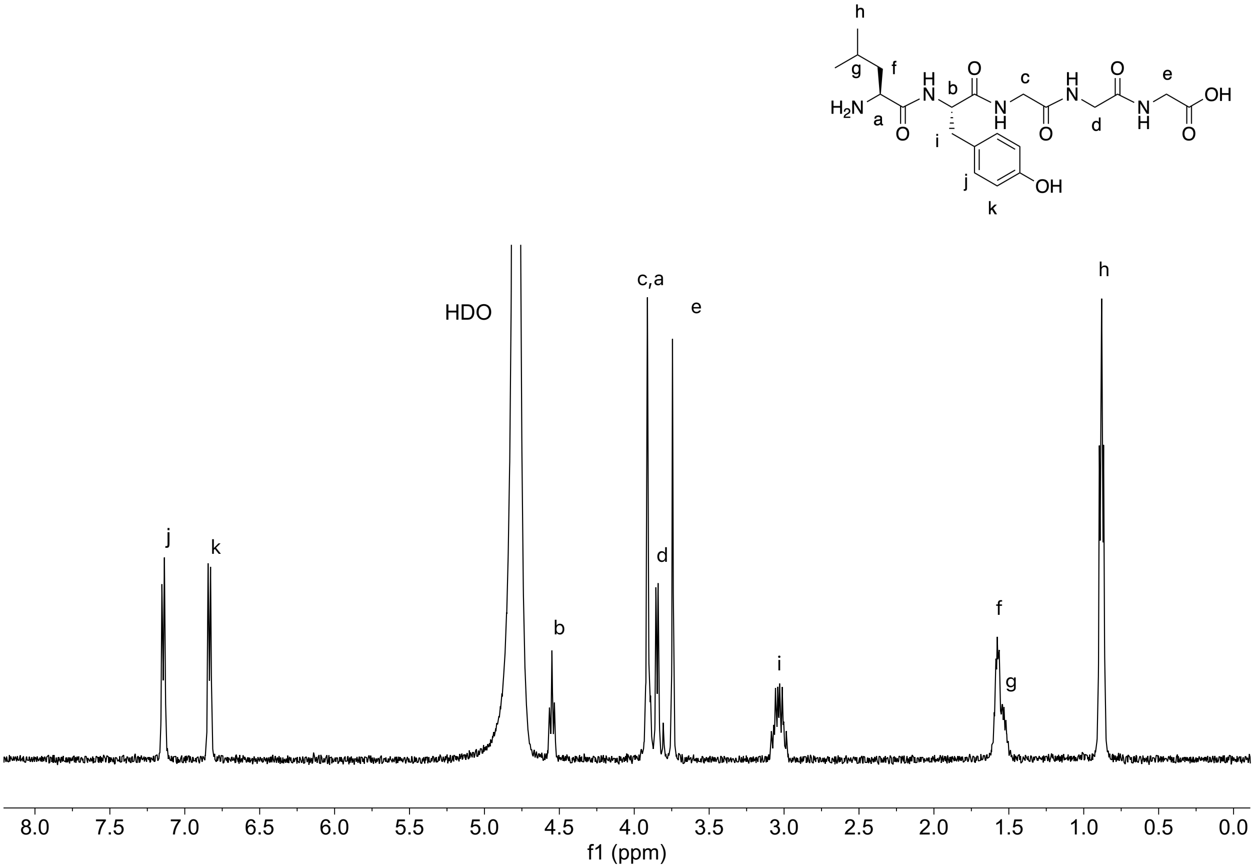

Figure S18: ^{1}H NMR spectrum (D_2O, 500 MHz, 25 °C) of H-LYGGG-OH.

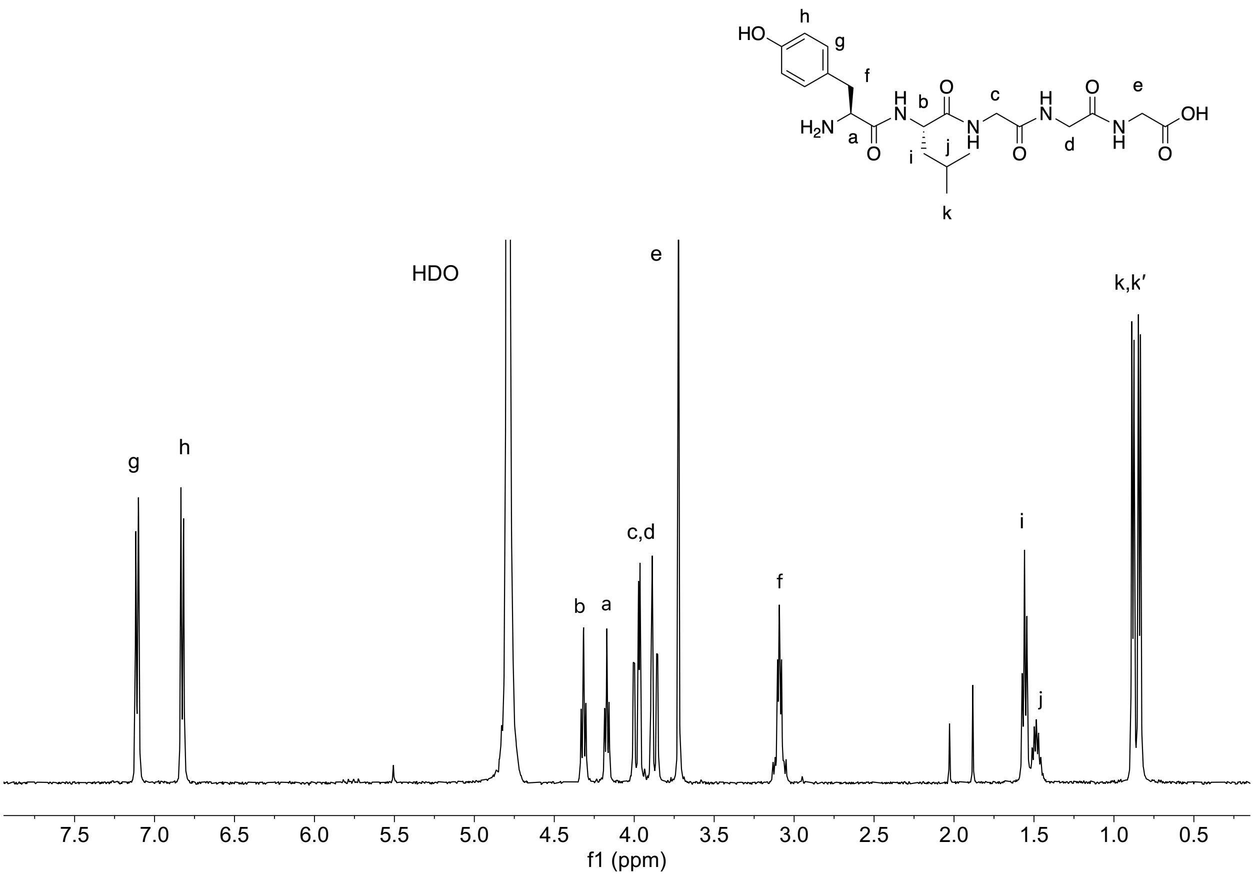

Figure S19: ^{1}H NMR spectrum (D_2O, 500 MHz, 25 °C) of H-YLGGG-OH.

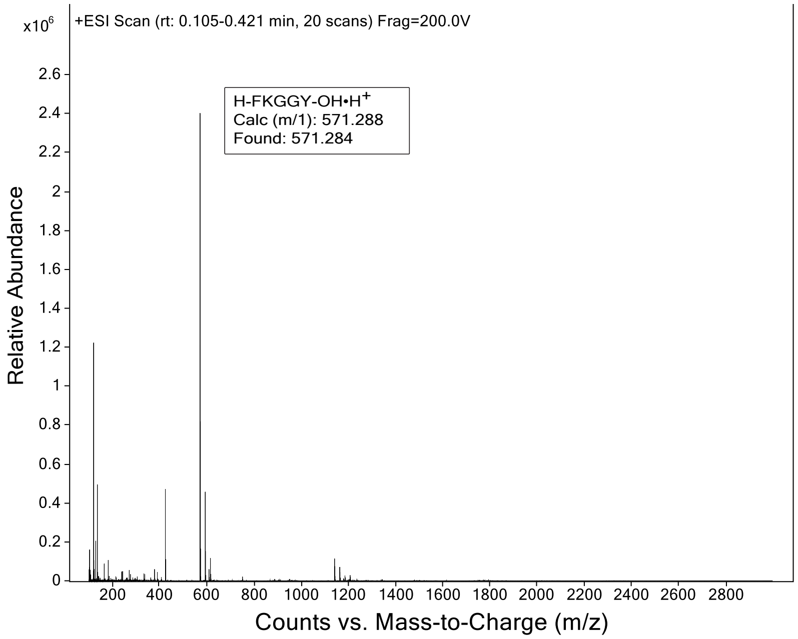

Figure S20: ESI-MS of a 100 μM aqueous solution of H-FKGGY-OH collected in positive mode.

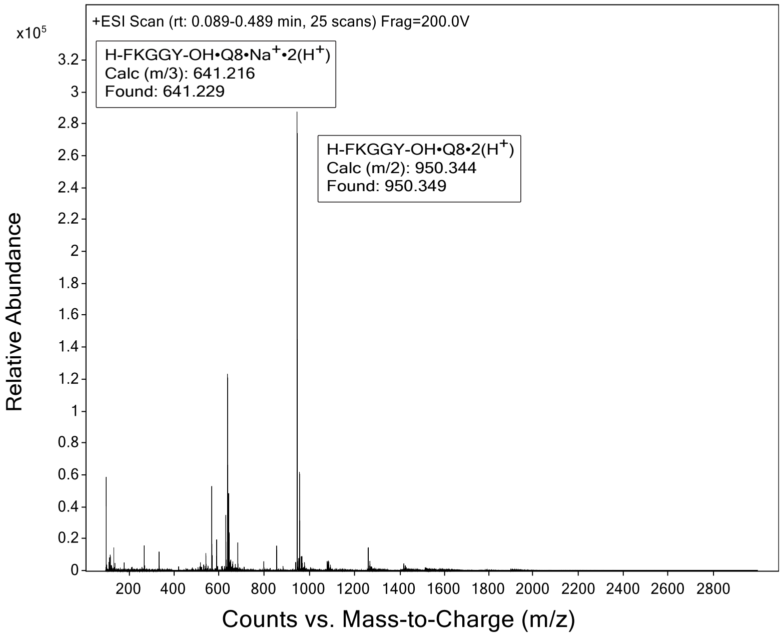

Figure S21: ESI-MS of a 100 μM aqueous solution of an equimolar mixture of H-FKGGY-OH and Q8 collected in positive mode.

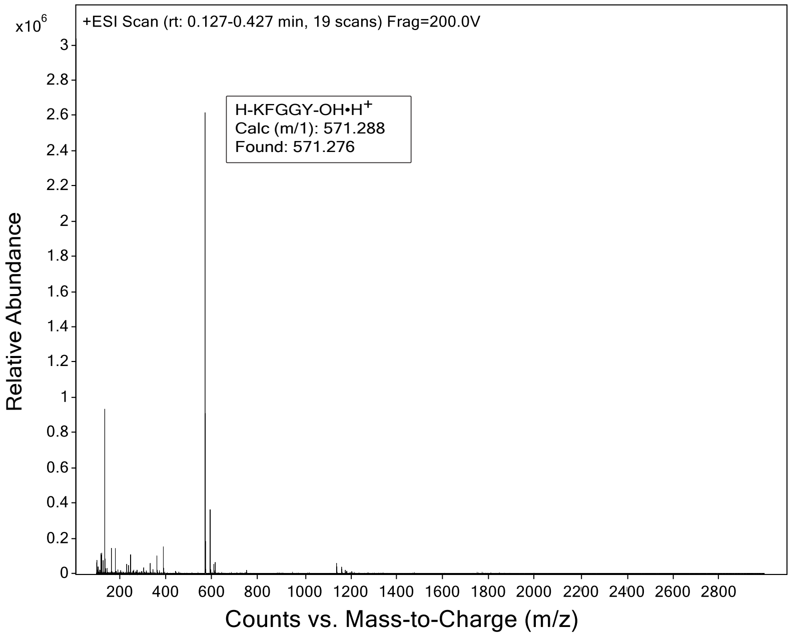

Figure S22: ESI-MS of a 100 μM aqueous solution of H-KFGGY-OH collected in positive mode.

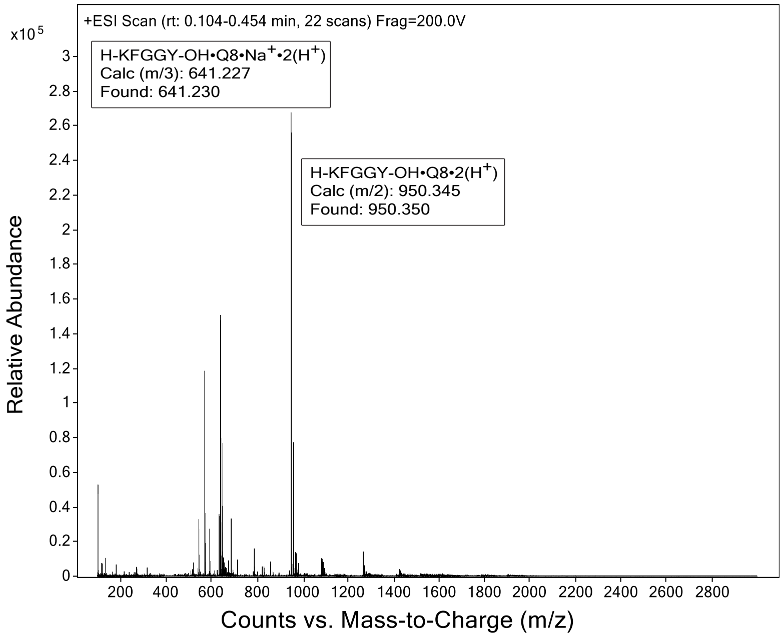

Figure S23: ESI-MS of a 100 μM aqueous solution of an equimolar mixture of H-KFGGY-OH and Q8 collected in positive mode.

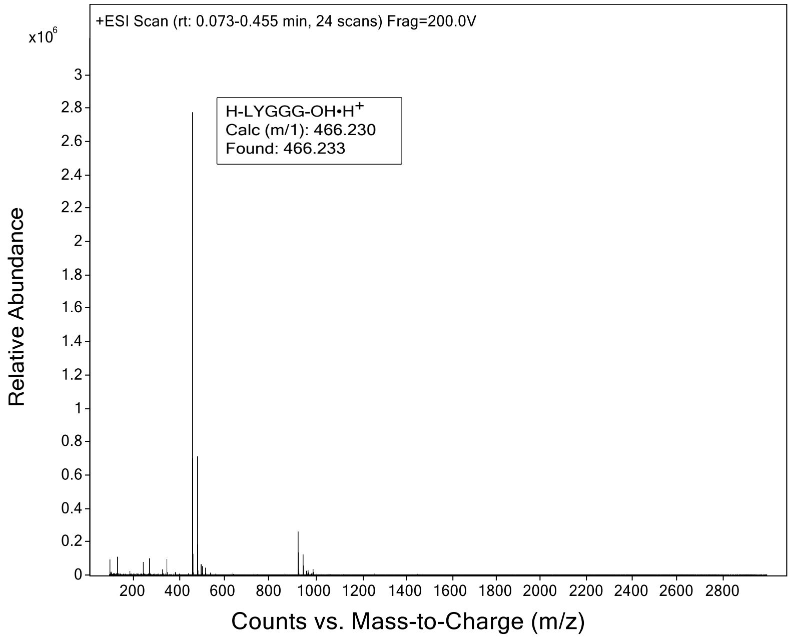

Figure S24: ESI-MS of a 100 μM aqueous solution of H-LYGGG-OH collected in positive mode.

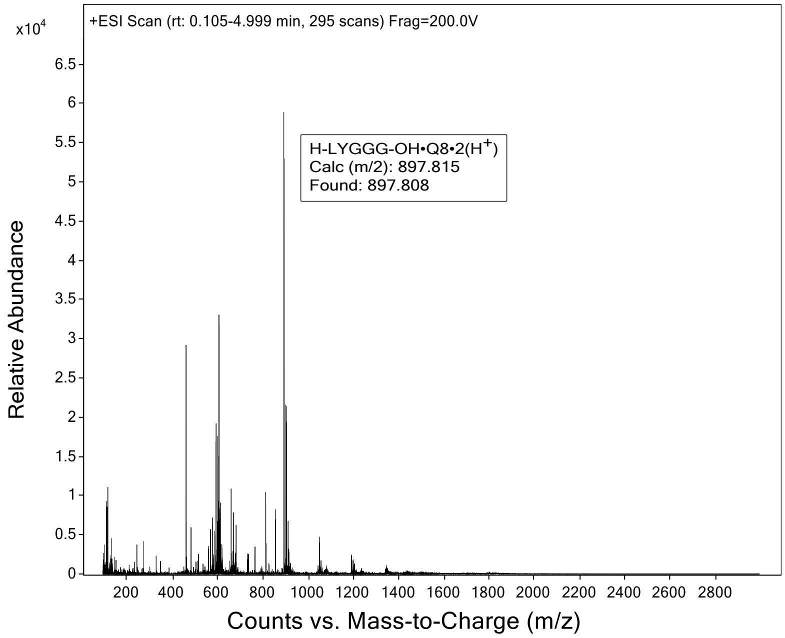

Figure S25: ESI-MS of a 100 μM aqueous solution of an equimolar mixture of H-LYGGG-OH and Q8 collected in positive mode.

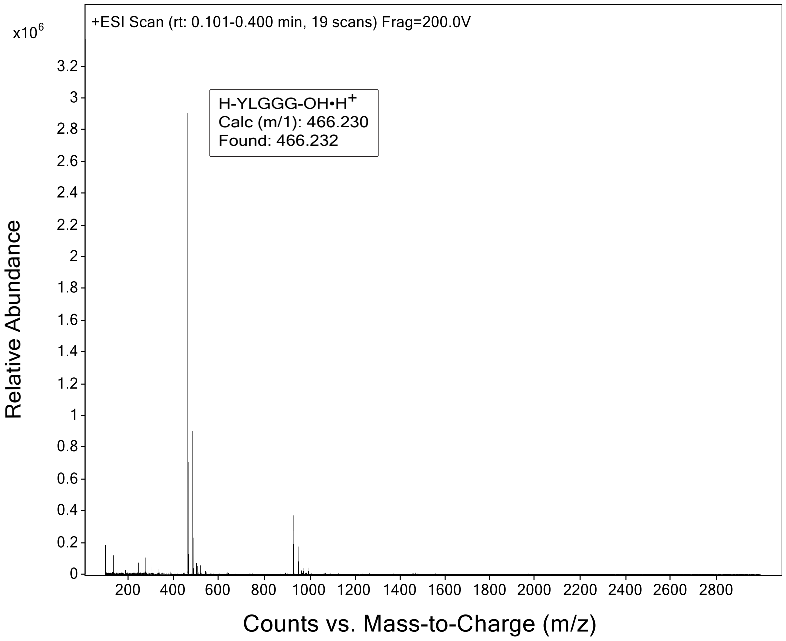

Figure S26: ESI-MS of a 100 μM aqueous solution of H-YLGGG-OH collected in positive mode.

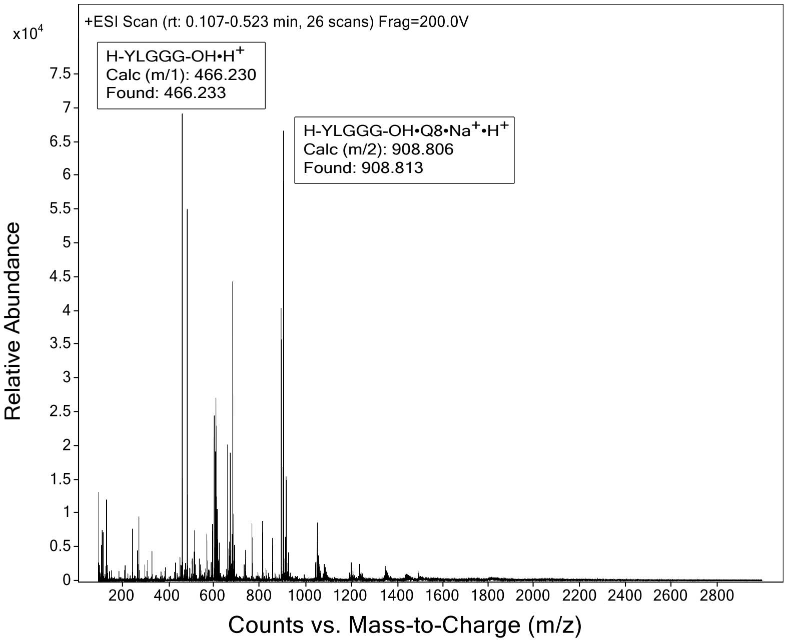

Figure S27: ESI-MS of a 100 μM aqueous solution of an equimolar mixture of H-YLGGG-OH and Q8 collected in positive mode.

References

1. A. Day, A. P. Arnold, R. J. Blanch and B. Snushall, Controlling Factors in the Synthesis of Cucurbituril and Its Homologues, *J. Org. Chem.*, 2001, **66**, 8094-8100. DOI: 10.1021/jo015897c
2. L. A. Logsdon, C. L. Schardon, V. Ramalingam, S. K. Kwee and A. R. Urbach, Nanomolar binding of peptides containing noncanonical amino acids by a synthetic receptor, *J. Am. Chem. Soc.*, 2011, **133**, 17087-17092. DOI: 10.1021/ja207825y
3. A. Krężel and W. Bal, A formula for correlating p*K*a values determined in D_2O and H_2O, *J. Inorg. Biochem.*, 2004, **98**, 161-166. DOI: 10.1016/j.jinorgbio.2003.10.001
4. S. Liu, C. Ruspic, P. Mukhopadhyay, S. Chakrabarti, P. Y. Zavalij and L. Isaacs, The cucurbit[n]uril family: prime components for self-sorting systems, *J. Am. Chem. Soc.*, 2005, **127**, 15959-15967. DOI: 10.1021/ja055013x
5. W. S. Jeon, K. Moon, S. H. Park, H. Chun, Y. H. Ko, J. Y. Lee, E. S. Lee, S. Samal, N. Selvapalam, M. V. Rekharsky, *et al.*, Complexation of ferrocene derivatives by the cucurbit[7]uril host: a comparative study of the cucurbituril and cyclodextrin host families, *J. Am. Chem. Soc.*, 2005, **127**, 12984-12989. DOI: 10.1021/ja052912c
6. Crysalispro 1.171.42.94a, 2023, Rigaku OD.
7. G. M. Sheldrick, Crystal structure refinement with SHELXL, *Acta Crystallogr., Sect. C: Cryst. Struct. Commun.*, 2015, **71**, 3-8. DOI: 10.1107/S2053229614024218
8. G. M. Sheldrick, SHELXT - integrated space-group and crystal-structure determination, *Acta Crystallogr., Sect. A: Found. Crystallogr.*, 2015, **71**, 3-8. DOI: 10.1107/S2053273314026370
9. O. V. Dolomanov, L. J. Bourhis, R. J. Gildea, J. A. K. Howard and H. Puschmann, OLEX2: a complete structure solution, refinement and analysis program, *J. Appl. Crystallogr.*, 2009, **42**, 339-341. DOI: 10.1107/s0021889808042726
